# The Evolution of Facultative Conformity Based on Similarity

**DOI:** 10.1371/journal.pone.0168551

**Published:** 2016-12-21

**Authors:** Charles Efferson, Rafael Lalive, Maria Paula Cacault, Deborah Kistler

**Affiliations:** 1 Department of Economics, University of Zurich, Zurich, Switzerland; 2 Department of Economics, University of Lausanne, Lausanne, Switzerland; University of Exeter, UNITED KINGDOM

## Abstract

Conformist social learning can have a pronounced impact on the cultural evolution of human societies, and it can shape both the genetic and cultural evolution of human social behavior more broadly. Conformist social learning is beneficial when the social learner and the demonstrators from whom she learns are similar in the sense that the same behavior is optimal for both. Otherwise, the social learner’s optimum is likely to be rare among demonstrators, and conformity is costly. The trade-off between these two situations has figured prominently in the longstanding debate about the evolution of conformity, but the importance of the trade-off can depend critically on the flexibility of one’s social learning strategy. We developed a gene-culture coevolutionary model that allows cognition to encode and process information about the similarity between naive learners and experienced demonstrators. Facultative social learning strategies that condition on perceived similarity evolve under certain circumstances. When this happens, facultative adjustments are often asymmetric. Asymmetric adjustments mean that the tendency to follow the majority when learners perceive demonstrators as similar is stronger than the tendency to follow the minority when learners perceive demonstrators as different. In an associated incentivized experiment, we found that social learners adjusted how they used social information based on perceived similarity, but adjustments were symmetric. The symmetry of adjustments completely eliminated the commonly assumed trade-off between cases in which learners and demonstrators share an optimum versus cases in which they do not. In a second experiment that maximized the potential for social learners to follow their preferred strategies, a few social learners exhibited an inclination to follow the majority. Most, however, did not respond systematically to social information. Additionally, in the complete absence of information about their similarity to demonstrators, social learners were unwilling to make assumptions about whether they shared an optimum with demonstrators. Instead, social learners simply ignored social information even though this was the only information available. Our results suggest that social cognition equips people to use conformity in a discriminating fashion that moderates the evolutionary trade-offs that would occur if conformist social learning was rigidly applied.

## Introduction

If people learn from others who vary, and if this learning process is not completely random, cultural evolution must occur in many situations. By extension, the psychology of social influence and social learning is almost certain to generate cultural evolutionary dynamics of one sort or another [1, 2]. Cultural evolution, in turn, can influence natural selection on genes, including genes that influence cognitive processes related to social influence and social learning. This is the basic logic of gene-culture coevolutionary theory, and it has been extremely influential in the evolutionary study of human behavior. It ties together, in an evolutionary process involving two linked systems of inheritance, the biological evolution of social cognition and the social cognition that underpins cultural evolution. People transmit and inherit both genetic and cultural information. Genes affect culture, and culture affects genes.

Conformist cultural transmission represents an especially prominent hypothesis. Conformity has a long history in the social sciences [3–8], and conformist cultural transmission is part of this tradition [9]. Conformist transmission centers around the hypothesis that the psychology of social learning generates a disproportionate tendency to follow the majority [1, 10, 11]. For example, if 75% of the people in a reference group exhibit a certain behavior, a conformist adopts this same behavior with a probability greater than 0.75. Such a tendency has the interesting property that it can stabilize differences between cultural groups [12–14] even in the face of persistent contact between the groups in question. Genes cannot do this, and for this reason conformity has figured repeatedly in the protracted, on-going, and acrimonious debate about the special role of culture in shaping the evolution of human cooperation [1, 2, 15–25].

Existing theory on the evolution of conformity typically assumes that frequency-dependent social learning strategies are fixed at the individual level [26, 27]. For example, if an agent has an inherited social learning strategy that dictates adopting the most common behavior in the social group, this response to frequency information does not vary for that agent. Conditional on social learning occurring, this assumption maximizes evolutionary trade-offs because the agent cannot adjust her use of social information according to circumstance, and for this reason the assumed rigidity of the social learning strategy is not simply an innocuous simplification for modelers. It is also not consistent with recent empirical evidence showing that the use of social information varies both across individuals and from one situation to another [27, 28].

We focus on how learners vary their response to social information from one situation to another because this kind of variation can radically affect evolutionary costs and benefits. Using both an evolutionary model and associated experiments, we specifically consider if and when social cognition supports a flexible response to information about the relative frequencies of different behaviors. To facilitate our discussion, we distinguish between two types of individual. Demonstrators have completed a learning process of some sort and made observable choices based on what they have learned. Learners, in contrast, are in the middle of their learning process. They are processing the information that will lead them to choose an observable behavior, but they have not yet made a choice. In particular, each learner observes the distribution of behaviors in a sample of demonstrators, and this social information somehow affects her own choice. In this sense, learners learn socially, and facultative adjustments to how learners do so will be our primary concern.

The adaptive value of conformist cultural transmission [1] involves two key considerations, namely the extent to which demonstrators choose their own optimum and the similarity between demonstrators and learners. Although demonstrators may not make optimal choices for various reasons [29, 30], demonstrators should exhibit some bias toward their own optimum if learning in the recent past has worked at all. If such a bias exists, the similarity between demonstrators and learners is decisive. Specifically, existing theory suggests that the evolution of conformity hinges critically on the rate at which learners and demonstrators are similar in that the same behavior is optimal for both [1, 26, 30–34].

Adopting the most common behavior among demonstrators will usually be beneficial for learners when demonstrators and learners have the same optimum, but detrimental when optima differ. Spatial and temporal heterogeneity [1, 31–36], specialized roles in society [33, 37, 38], recent technological innovations [29, 39, 40], and transmission errors [28, 33] can all affect the probability that a learner learns from demonstrators who themselves learned under similar circumstances. The details matter. For example, temporal heterogeneity should expose learners to demonstrators with a different optimum if cultural transmission is across generations, but not if transmission is within generations. Analogously, spatial heterogeneity should expose learners to demonstrators with a different optimum if demonstrators learned in one location, only to move around and demonstrate their learned behaviors in other locations. However, if the sequence is move, then learn and demonstrate, any discrepancy in optima between learners and demonstrators should be less pronounced.

The common theme here is that some set of mechanisms produces a probabilistic relationship between demonstrator optima and learner optima. Whatever the underlying mechanisms, this relationship affects the costs and benefits of different social learning strategies. If a conformist bias evolves under positive covariance in optima, for example, and if the bias is indiscriminately applied by social learners, it will bring a benefit when learners and demonstrators are similar and a cost when different. Natural selection must sort out the relative importance of these costs and benefits, and theory about the evolution of conformist transmission generally takes this form [26, 27, 33, 34]. The calculus changes, however, if learners can adjust how they use social information according to circumstance.

We developed and analyzed a gene-culture coevolutionary model (S1 Appendix) in which learners, apart from processing information about the distribution of behaviors among demonstrators, can also process information about their similarity to demonstrators. In practice, we suspect that information about similarity can take many forms. In some cases, the information may directly address the relevant question of whether learners and demonstrators share an optimum. Charles admires LeBron James, but even a cursory comparison directly reveals that in many domains Charles’ optimal choices are not the same as Mr. James. In other cases, information about similarity could be something like a shared ethnic marker [41] that correlates positively with learners and demonstrators having the same optimum.

Whatever form information about similarity might take, we show that under the right conditions social cognition evolves to support facultative social learning strategies that condition on perceived similarity. For this to happen, learners must be exposed to demonstrators with different optima at a sufficiently high rate, and they must have sufficiently reliable information about similarity. Interestingly, when facultative social learning strategies evolve, adjustments often evolve to be asymmetric. This means the response to social information is most extreme when a learner has information indicating she is similar to demonstrators. The response is less extreme when available information indicates dissimilarity.

We examined these ideas with an experiment. We tested for both facultative social learning strategies and, if facultative, asymmetries in the adjustments participants make. We then followed up with a second experiment. This second experiment allowed us to see if social learners reveal preferences for specific forms of social learning, and if they reveal assumptions that reflect an evolutionary history in which demonstrators and learners usually shared an optimum. In particular, if social cognition evolved under conditions in which social learners were typically similar to those from whom they learned, and if cognition does not fully adjust to departures from these typical conditions in the past, then social learners should have some preference for following the majority and some tendency to assume similarity. Our second experiment tested these ideas by maximizing the potential for social learners to reveal both their preferred social learning strategies and any assumptions they might make about their similarity to demonstrators. Put differently, we asked if a preference for conformity and an assumption of similarity might be part of a core system of social knowledge [42]. Our empirical strategy for this question was to remove all incentives and sources of information that might conflict with such a core system and observe what remained.

## The evolved social cognition of perceived similarity

Consider a setting with two environmental states and two behaviors. Behavior 0 is optimal in state 0. Behavior 1 is optimal in state 1. We examine the evolution of cognition and an associated learning system in this setting (see S1 Appendix for details). Demonstrators have completed their learning process and chosen either 0 or 1. This choice is observable to others. Learners, in contrast, are in the middle of their learning process. They are processing the information that will lead them to choose an observable behavior, but they have not yet made a choice. Learners do not know which state they face, but they enjoy high payoffs if they choose optimally. This provides the basis for the evolution of cognition. Given time, natural selection identifies the cognitive systems best at leading learners to choose optimally and receive high payoffs as a result.

### A cognitive system that ignores similarity

Before choosing, each learner receives two types of information. In no particular order, each learner receives a private signal, s∈R. This signal provides noisy information about the state the learner faces. Each learner also samples *N* demonstrators and observes that *i* of them exhibit behavior 1. In effect, by observing *s* the learner learns individually because she considers her own private information about the environmental state. By observing *i* the learner learns socially because she considers the choices of others. The state among demonstrators is *y*, and the state among learners is *z*. Demonstrators and learners face a different state and thus have different optima with probability *γ*. They face the same state and have the same optimum with probability 1 − *γ*. We will routinely refer to the former situation as “discordant” and the latter as “concordant.” Ex ante, the covariance between learner and demonstrator optima is (1 − 2*γ*)/4. To summarize the learner’s problem intuitively, the learner has some private information (*s*), she has some social information (*i*), and she needs to make a choice consistent with her environment (*z*).

Perreault and colleagues [34] developed an innovative approach to modeling the evolution of a learning system in this setting. They did not simply posit a set of learning strategies according to what seems reasonable to the modeler and then let selection sort out the best strategies. Rather, they assumed that learners need to solve an inferential problem based on the Bayesian posterior, *P*(*z*|*i*, *s*). The posterior is used by the modeler to identify the information a decision maker would need to draw a Bayesian inference. The learners under consideration, however, do not actually make Bayesian calculations. Rather, they have cognitive representations of the information required for an inference. These cognitive representations are inherited genetically, they can vary across learners, and they summarize the stochastic properties of the decision-making task in a more or less accurate way. For a given learner, the cognitive representation is fixed, and it consists of two quantities. The first quantity summarizes how reliably *s* identifies the learner’s environment, and hence it is a representation of how effective individual learning is. We use the symbol $\hat{\alpha }$ for this quantity (S1 Appendix). The second quantity summarizes how effectively demonstrators choose their own optimum, regardless of whether learners and demonstrators have the same optimum. We use $\hat{q}$ for this quantity (S1 Appendix). In the model of Perreault and colleagues [34], these two quantities enter the model as a single unit-free combination, and this combination is the one-dimensional trait under selection.

In particular, given an inherited value for this trait, a learner observes *i* and *s* and then chooses a behavior by following a simple rule that would maximize expected payoffs under a Bayesian posterior. The learner, however, does not necessarily maximize expected payoffs because her cognitive representation of the decision-making task does not necessarily produce the same posterior as an actual Bayesian.

Evolution proceeds as follows [34]. Genetically inherited cognitive representations affect how learners choose between the two possible behaviors. Learning can lead the distribution of behaviors in a population to change through time in a cultural evolutionary process. As the distribution of behaviors changes, the relative values of different cognitive representations can also change. Natural selection responds accordingly in a genetic evolutionary process. The distribution of behaviors and the distribution of cognitive representations evolve together in a gene-culture coevolutionary system. A learning system that integrates private and social information emerges endogenously and eventually stabilizes.

The steady-state learning system has two crucial features [34]. First, it optimizes the trade-off between the accuracy of individual learning and the economy of social learning [43, 44]. Second, learners exhibit positive social influence. Positive social influence means that, as the observed frequency of a behavior increases among demonstrators, the probability that a learner chooses the same behavior also increases. Negative social influence means the opposite; learner choices are negatively related to demonstrator choices. Crucially, not all forms of positive social influence are equivalent in terms of the cultural evolutionary dynamics they produce [11]. A learning system based on *P*(*z*|*i*, *s*), however, often evolves to show the characteristic “S” shape of conformist cultural transmission [34]. Because equilibrium strategies optimize the trade-off between private and social information, individual learning can reduce the expected costs of conformity when the learner optimum is at low frequency among demonstrators. The extent to which this holds, however, depends on the reliability of private information (see [34] and S1 Appendix).

In any case, because the evolution of cognition is based on *P*(*z*|*i*, *s*), which only considers the learner’s state, cognition does not allow learners to process information about the similarity between themselves and the demonstrators from whom they learn. This imposes the strongest possible evolutionary trade-off between discordant and concordant instances. Conformity, or positive social influence more broadly, is costly in the former case and advantageous in the latter. If learners, however, had some idea about whether they share an optimum with demonstrators, learners could potentially retain the upside of conformity and attenuate the downside. In particular, they could do so more than what is possible by simply relying on individual learning to limit the occasional damage caused by conformity.

### A cognitive system that considers similarity

To examine how evolution might attenuate the trade-off between discordant and concordant instances, we developed a model (S1 Appendix) that allows cognition to process information about the similarity between demonstrators and learners. In addition to *i* and *s*, each learner observes a noisy private signal, *a*, that indicates whether she and the demonstrators she learns from have the same optimum. As mentioned earlier, this signal may pertain directly to the question of whether a learner and her demonstrators have the same optimum. However, it might also be some other, potentially correlated variable like sex, ethnicity, or dialect. Whatever the underlying mechanism, the signal of similarity is correct with probability *ϕ* ≥ 0.5. Perceived similarity modulates how the learner responds to social information. Because we allow cognition to evaluate similarity, we consider the evolution of an inferential system based on the Bayesian posterior *P*(*y*, *z*|*a*, *i*, *s*). In other words, learners do not simply consider the state they face (*z*); they also consider the state demonstrators faced when demonstrators were themselves learners (*y*).

Learners do not actually perform Bayesian calculations. Rather, we use the Bayesian posterior to identify the structure of the cognitive system that evolves under natural selection. In particular, the model outlined earlier [34] involves a single cognitive representation that is a unit-free combination of $\hat{\alpha }$ and $\hat{q}$. Aside from $\hat{\alpha }$ and $\hat{q}$, our model requires two additional quantities (S1 Appendix). The quantity $\hat{\gamma }$ is the cognitive representation of the probability that learners and demonstrators have different optima. It is, in effect, an inherited Bayesian prior pertaining to similarity. The quantity $\hat{\phi }$ is the cognitive representation of the ex ante probability that the signal of similarity (*a*) is correct. This inherited representation affects how the learner responds to a realized signal of similarity. Importantly, *γ* and *ϕ* capture the actual structure of the decision-making task; they do not change. In contrast, $\hat{\gamma }$ and $\hat{\phi }$ are genetically inherited cognitive representations of these same quantities. They may be accurate for one individual, but they can also be wildly inaccurate for another. $\hat{\gamma }$ and $\hat{\phi }$ do not change at the individual level, but the distribution of $\hat{\gamma }$ and $\hat{\phi }$ values in the population changes through time as the evolutionary process unfolds.

We show (S1 Appendix) that a gene-culture coevolutionary system based on *P*(*y*, *z*|*a*, *i*, *s*) gives rise to learning strategies that are sometimes, though not always, facultative. If a strategy is facultative, the learner conditions how she learns on her perceived similarity to demonstrators (i.e. *a*). To motivate our experiments, we now focus on three key findings from the model. These findings hold for cases in which individual learning is relatively difficult. We focus on these cases because, when individual learning is difficult, evolution leads to learning systems that emphasize social learning. An emphasis on social learning, in turn, ensures scope for the evolution of a cognitive system that responds to perceived similarity by changing how learners learn from demonstrators. As explained later, we examined an extreme version of this scenario in our experiments by forcing a subset of participants to learn exclusively via social learning.

When individual learning is relatively difficult, which is equivalent to saying that *s* is a relatively noisy indicator of the learner’s state, three key findings follow.

**Fig 1:**
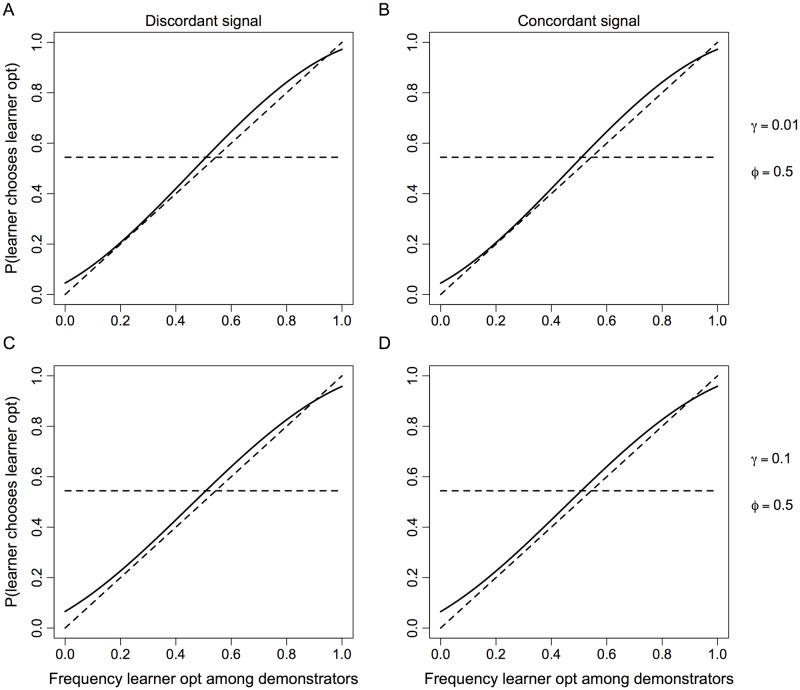
Evolved learning strategies when individual learning is relatively unreliable and the signal of similarity is uninformative. Solid lines summarize the properties of the learning system by showing the probability that learners choose their own optimum as a function of how common this same behavior is among demonstrators. Learning strategies potentially depend on whether a learner receives a signal of similarity indicating either different optima (Discordant signal, **A** and **C**) for learners and demonstrators or the same optimum (Concordant signal, **B** and **D**). This signal of similarity is uninformative (*ϕ* = 0.5, cf. Fig 2). Rows vary according to whether a difference in optima is rare (**A** and **B**, *γ* = 0.01) or more common (**C** and **D**, *γ* = 0.1). The horizontal dashed lines show a learning system that ignores demonstrator behavior and relies only on individual learning. The diagonal dashed lines show an unbiased learning system that does not generate cultural evolution. See S1 Appendix for model details.

**Fig 2:**
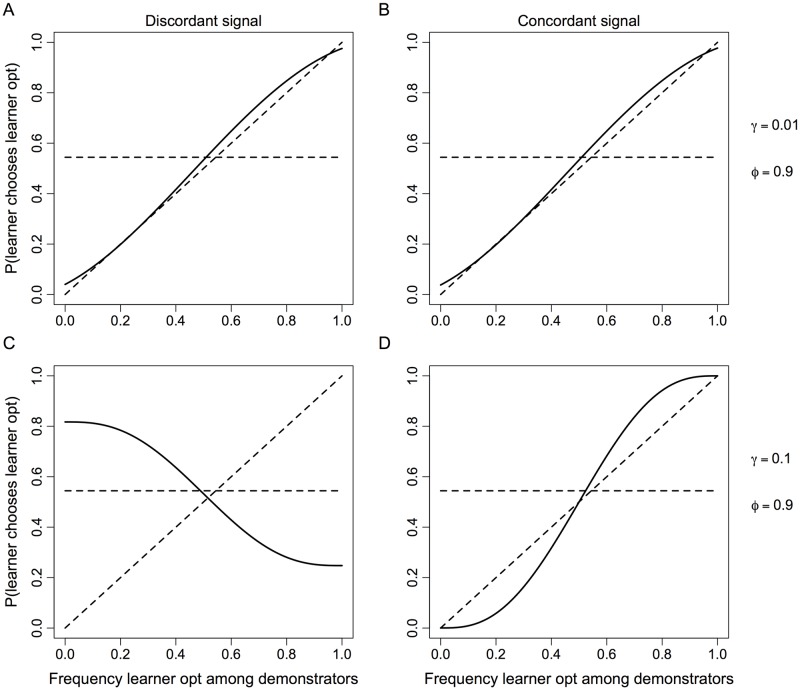
Evolved learning strategies when individual learning is relatively unreliable and the signal of similarity is informative. Solid lines summarize the properties of the learning system by showing the probability that learners choose their own optimum as a function of how common this same behavior is among demonstrators. Learning strategies potentially depend on whether a learner receives a signal of similarity indicating either different optima (Discordant signal, **A** and **C**) for learners and demonstrators or the same optimum (Concordant signal, **B** and **D**). This signal of similarity is informative (*ϕ* = 0.9, cf. Fig 1). Rows vary according to whether a difference in optima is rare (**A** and **B**, *γ* = 0.01) or more common (**C** and **D**, *γ* = 0.1). The horizontal dashed lines show a learning system that ignores demonstrator behavior and relies only on individual learning. The diagonal dashed lines show an unbiased learning system that does not generate cultural evolution. See S1 Appendix for model details.

1. If the signal of similarity is uninformative (*ϕ* = 0.5), the evolved learning system is not facultative, and it typically exhibits positive social influence (Fig 1). These results are intuitive. Without a meaningful indicator of similarity, cognition does not evolve to condition learning on perceived similarity. Positive social influence evolves because we typically assume learner optima covary positively, though to varying degrees, with demonstrator optima. Natural selection simply responds to the standard trade-off between discordant and concordant cases. Because concordance is common, positive social influence evolves as it would without a signal of similarity.
2. Facultative learning evolves if learners benefit from discriminating based on similarity (*γ* > 0), and they have information that allows them to do so (*ϕ* > 0.5). The joint effect of these two mechanisms is critical. For example, if learners and demonstrators almost always have the same optimum (e.g. 1 − *γ* = 0.99), the evolved learning system may not be meaningfully facultative (Fig 2) even if the signal of similarity is quite reliable (e.g. *ϕ* = 0.9). However, holding signal reliability constant, the need to discriminate based on similarity increases as the positive covariance in optima declines (e.g. 1 − *γ* = 0.9), and the learning system that evolves is facultative (S1 Appendix). Moreover, if discriminating is sufficiently important and the signal of similarity is sufficiently reliable, the learning system can evolve to be strongly facultative (Fig 2). By strongly facultative we mean that learners follow the minority choice among demonstrators if the signal of similarity indicates different optima, and they follow the majority choice if the signal indicates the same optimum. The former is a form of negative social influence and the latter a form of positive social influence. In other cases (e.g. 1 − *γ* = 0.9 and *ϕ* = 0.7), learning can evolve to be facultative but simply exhibit varying degrees of positive social influence.
3. Facultative adjustments are often asymmetric. As explained in the previous point, evolution can produce learners who switch, based on perceived similarity, between following a minority of demonstrators and a majority of demonstrators. Facultative switching of this sort is often asymmetric. This means the tendency to follow the minority when the signal of similarity indicates different optima is weaker than the tendency to follow the majority when the signal indicates the same optimum (Fig 2). Asymmetric learning systems tend to evolve when a shared optimum is typical. A Bayesian analysis clarifies why this is important (S2 Appendix). If a shared optimum is typical (*γ* < 0.5), we can view 1 − *γ* > 0.5 as a prior biased in favor of a shared optimum. An informative signal of similarity (*ϕ* > 0.5) that takes a realized value indicating a shared optimum reinforces this biased prior and leads to an even more strongly biased posterior. In contrast, a realized signal indicating different optima must first offset the biased prior before it can support a biased posterior favoring a belief in different optima. This is the difference between a signal indicating similarity and a signal indicating dissimilarity. One can reduce the asymmetry, but this requires the covariance in optima to approach zero or signals of similarity to approach perfect reliability (S2 Appendix). Otherwise, Bayesian posteriors are inevitably asymmetric. Results from our evolutionary model (S1 Appendix) show analogous patterns. For example, a moderately informative signal (*ϕ* = 0.7) leads to the evolution of symmetric adjustments (S2 Appendix) when learner optima do not covary with demonstrator optima (1 − *γ* = 0.5). Under positive covariance (e.g. 1 − *γ* = 0.75), however, the evolved learning system produces highly asymmetric adjustments under the same signal reliability (S2 Appendix). One can reduce the asymmetry, but this requires an increasingly reliable signal of similarity (e.g. *ϕ* = 0.9, S2 Appendix).

We conducted an experimental study to examine these ideas. Overall, we varied both the similarity between demonstrators and learners and the information learners had about similarity. The objective was to understand the flexibility of social learning strategies and how the degree of flexibility affects payoffs. For example, if frequency-dependent social learning only responds to changes in the distribution of choices among demonstrators, none of our experimental manipulations should have mattered. If our manipulations did matter, however, this would generically indicate that social learning can also depend on perceived similarity. Our model would further suggest the following in this case. If social cognition evolved under positive covariance in optima and signals of similarity that were not excessively reliable, and if contemporary social cognition reflects these past regularities, we expect asymmetric facultative adjustments. Otherwise, either a shared optimum was not typical in the past, signals of similarity were exceedingly reliable, or contemporary social cognition is subtle enough to make adjustments beyond those accounted for in our model.

## Experiment 1

We divided participants into demonstrators, who learned individually, and social learners, who could only learn by observing demonstrators. We use the term “social learner” here to refer simply to a participant’s role in the experiment. We do not mean to imply that social learners necessarily used the social information available to them. We implemented both discordant and concordant scenarios, and we provided social learners with information to infer which of the two scenarios they were facing. Existing theory on the evolution of conformity assumes social learning strategies that do not discriminate based on this kind of information. If this assumption is generally valid, we should find that the social learners in our experiment did not discriminate. If they did discriminate, social learning was facultative. We have shown that in theory facultative strategies often evolve to be asymmetric. As we explain below, asymmetric adjustments in our experiment would have necessarily reduced social learner payoffs in either discordant or concordant situations, and we exploited this fact to test for asymmetries.

### Materials and methods

For each decision-making trial, a participant had to choose between two urns, one on the “left” and the other on the “right.” The urns contained red and blue balls, and participants received payments for balls of one color but not the other. Specifically, one urn contained one blue ball and three red balls, while the other contained one red ball and three blue balls. After choosing an urn, a ball would be randomly drawn from the chosen urn and then replaced. If the ball was the winning color, the participant would receive 100 points. If the ball was the other color, the participant would receive zero points. Each subject received points based only on her own choices. Points were later converted to cash as payments made privately at the end of a session. Like the model explained above, each participant had to choose between two options. One option was optimal because it had more balls of the winning color. The other option was sub-optimal.

At the beginning of a session, we divided all participants into two groups. The participants in these two groups did not interact in any way during the session. Within a group, we randomly assigned five participants to be demonstrators and from four to seven participants to be social learners. Demonstrators only had private information about the consequences of their own choices, while social learners only had public information about the choices of demonstrators. We separated individual and social learning in this way for two reasons. First, our focus is on the facultative use of social information. If participants in an experiment learn via both private and social information, learning can potentially be facultative with respect to both types of information. This would complicate the task of identifying how social learning in particular changes, and we eliminated this possibility by making demonstrators and social learners two distinct roles.

Second, if experimental subjects engage in repeated bouts of social learning, with subjects repeatedly choosing and observing each other’s choices, estimates of social influence are biased because of the “reflection problem” [45, 46]. The reflection problem is analogous to inferring causality when a researcher looks at herself in the mirror and waves. Given the evidence, she cannot say if her hand is moving her reflection or if her reflection is moving her hand. Similarly, if a subject repeatedly makes observable choices in a group of people who are also making observable choices, the researcher cannot readily isolate the causal effect of social information on the choices of a focal subject. The researcher can posit that decision making has a specific temporal structure, but this can require strong and potentially groundless assumptions. An experimental design that avoids reflection altogether may thus be preferable. We ensured that reflection was not possible by allowing demonstrators to engage in multiple bouts of individual learning, followed by a single bout of social learning among social learners. In effect, demonstrators produced social information, and social learners consumed it. Participants did not change roles. Subjects made decisions in 20 independent blocks of five trials per block.

To illustrate procedures in a concrete way, assume that red was the winning color for demonstrators. At the beginning of a block of five trials, the two sets of balls were randomly assigned to the urns for all subjects in a group, both demonstrators and social learners. The urn with three red balls was optimal for demonstrators in the sense that the expected payoff from choosing this urn was 75 points. Demonstrators chose repeatedly and could learn which urn was optimal for them by seeing the color of the ball drawn after each choice. Demonstrators had no other information, and thus learning was strictly individual.

The social learners in a group faced the same two urns as the demonstrators. Each social learner, however, only made a single choice per block. After all demonstrators had chosen an urn in the fifth trial of a block, the distribution of urn choices among the demonstrators, for the fifth trial only, was communicated to the social learners. Each social learner then chose an urn. Five balls were randomly drawn with replacement from the chosen urn to determine payoffs. We made five draws per choice for each social learner so that both demonstrators and social learners would receive the same number of realized payoffs. Social learners did not receive information about their realized payoffs at the end of each block. Instead, they only received aggregated feedback at the end of the session, and for this reason they could not learn how to best use social information as the session progressed.

We implemented four treatments that varied in terms of the relationship between demonstrators and social learners. In **discordant** blocks, demonstrators and social learners received points for balls of different colors. **Concordant** blocks were the opposite; demonstrators and social learners both received 100 points for the same color of ball. The discordant-concordant distinction was implemented within social learners in some sessions. This means that each social learner participated in 10 discordant blocks and 10 concordant blocks in an alternating fashion. To avoid anchoring effects, we counterbalanced the initial block across the two groups of participants within sessions. Implementing both discordant and concordant blocks within subjects should have made the difference relatively salient. We implicitly invited social learners to consider the difference and recognize, if they cared to, that they might want to change their use of social information from one block to the next.

In other sessions, the discordant-concordant distinction was implemented between social learners. Accordingly, each social learner participated in either 20 discordant blocks or 20 concordant blocks. To avoid session effects, we always had one group of discordant participants in a session and another group of concordant participants. In these between-subjects treatments, the discordant-concordant distinction was not salient. Indeed, social learners in a discordant group were not informed about the presence of concordant groups and vice versa. Social learners were not implicitly invited to consider different ways of using social information by explicitly exposing them to both discordant and concordant treatments. The design of experiment 1 is two-by-two. The similarity between demonstrators and social learners was either discordant or concordant, and this treatment variation was implemented either within social learners or between social learners. In all four cases, social learners were told whether they were in a discordant or concordant block, and so we call these treatments “transparent.”

Importantly, the ex ante probability that a social learner and her demonstrators had the same optimum was 0.5, and thus the covariance in optima was zero. In addition, the signal was correct with probability one. This does not mean, however, that we necessarily expect social learning strategies consistent with these values. Our model does not allow learners to evaluate current values of *γ* and *ϕ*. Rather, learners have inherited values of $\hat{\gamma }$ and $\hat{\phi }$ that evolved under *past* values of *γ* and *ϕ*. This is a fundamental point about the extent to which we expect social learning strategies to reflect past regularities, and we discuss it at length in the general discussion.

To avoid uninteresting experimental artifacts related to spatial biases or color preferences, we counterbalanced the color of ball producing 100 points for demonstrators across sessions. In addition, when choosing an urn, subjects clicked a button labeled “left” or “right.” These buttons were not oriented left to right, but rather top to bottom. The urn listed on top was randomly determined for each subject and each trial.

We conducted seven sessions using z-Tree [47] under anonymous laboratory conditions at the University of Lausanne. Our data include observations from 70 demonstrators and 76 social learners. Each participant received a show-up fee of 10 Swiss Francs, and the average total payment was 41.38 Swiss Francs. Before participating, participants provided informed consent by signing a consent form. The consent procedure and methods for the entire study were approved by the Human Subjects Committee of the Faculty of Business and Economics at the University of Lausanne and by the Human Subjects Committee of the Faculty of Economics, Business Administration, and Information Technology at the University of Zurich. Select screen shots from the z-Tree program are presented in S3 Appendix, translated instructions are available as S1 Instructions, and raw data are available as S1 Data.

### Results

Demonstrators identified their own optimal urns effectively. Demonstrators chose their optima in the final periods of blocks at a rate well above chance (logistic regression, robust standard errors clustered on demonstrator, *p* ≪ 0.001). Of the five demonstrators in a group, a majority chose the demonstrator optimum in the final period of a block in 90.7% of all blocks. Four out of five choosing optimally was the modal outcome. This result means that, in the final period of discordant blocks, the minority choice among demonstrators was typically the optimal choice for social learners. In concordant blocks, the majority choice was typically the optimal choice for social learners.

**Fig 3:**
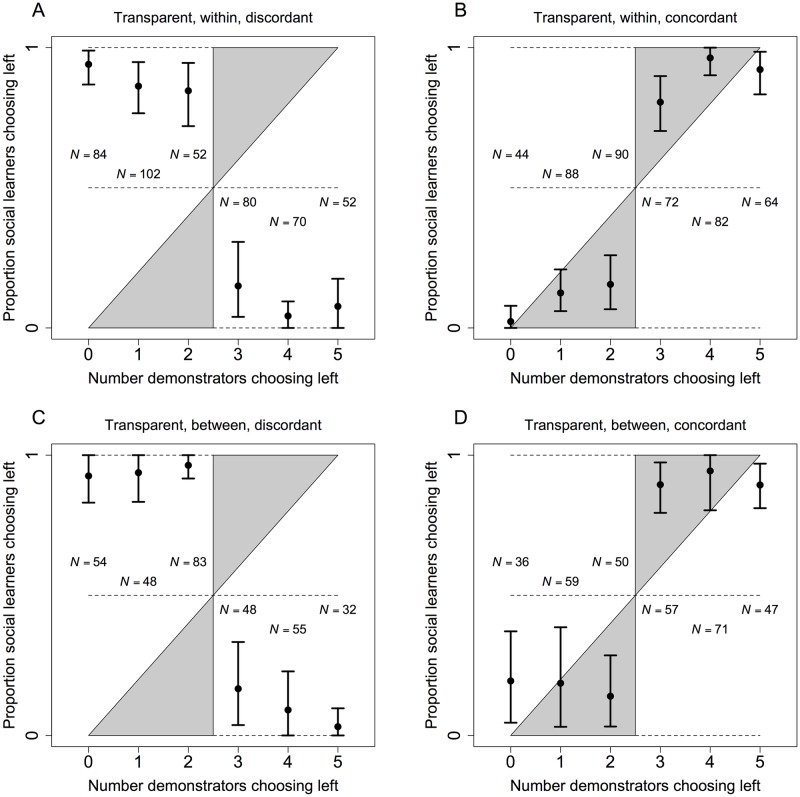
Left choices by social learners in the four transparent treatments from experiment 1. The points show the rate at which social learners chose left as a function of the number of demonstrators choosing left in the final trials of blocks. The number of observations is vertically aligned with each point. Panels **A** and **C** show the discordant treatments, while panels **B** and **D** show the concordant treatments. Panels **A** and **B** show the within-subjects treatments, while panels **C** and **D** show the between-subjects treatments. Error bars are 95% bootstrapped confidence intervals clustered on social learner. Clustering accounts for correlations due to multiple observations per social learner [48], and bootstrapping ensures that confidence intervals remain in [0, 1]. The gray region of the space is consistent with conformist cultural transmission [1, 11]. The diagonal in black is consistent with unbiased social learning, which does not generate cultural evolution, and dashed lines provide additional points of reference at 0, 0.5, and 1.

Fig 3 shows the average social learning functions by treatment. Social learners exhibited a strong tendency to follow the minority choice among demonstrators in discordant blocks and the majority choice in concordant blocks. Table 1 shows results from associated logistic regressions. The key finding is that the response to social information was strongly facultative. For discordant blocks, social learners showed a highly significant decrease in the rate of choosing left as the number of left choices among demonstrators increased (Table 1, “Prop left, centered”). In concordant blocks, the result was the opposite, with social learners showing a highly significant increase in choosing left in tandem with demonstrators (Table 1, “Prop left, centered”). In all cases, social learning functions are close to the boundaries at zero and one, with a sharp discontinuity between two and three demonstrators choosing left, and this indicates limited heterogeneity among social learners (Fig 3)

**Table 1:** Social learners choosing left in discordant and concordant treatments from experiment 1. Results are from logistic regressions. Independent variables include an index for the 20 learning blocks in each session and the distribution of choices among demonstrators in the fifth trial of the block. This distribution is centered at zero. Thus, if *p*_5_ is the proportion of demonstrators choosing left in the fifth trial, we transform this as *x*_5_ = *p*_5_ − 0.5. Robust standard errors clustered on social learner account for any correlations due to multiple observations per social learner [48, 49], and we show these robust clustered standard errors in parentheses.

Parameter	Within-subjects	Between-subjects
	Discordant	
Intercept	-0.849 (0.444)	0.707** (0.259)
Learning block	0.048 (0.026)	-0.029 (0.027)
Prop left, centered	-7.446*** (1.432)	-9.163** (2.952)
	Concordant	
Intercept	0.358 (0.229)	0.156 (0.232)
Learning block	-0.040 (0.023)	0.018 (0.021)
Prop left, centered	8.335*** (1.363)	5.867*** (1.495)

*** (0.001)

** (0.01)

* (0.05)

As discussed earlier, our model shows that social cognition often evolves to produce asymmetric facultative adjustments. To test for asymmetry, we analyzed the rate at which social learners chose the social learner optimum as a function of whether a majority of demonstrators chose the demonstrator optimum (Table 2). The results reveal no trace of an asymmetry. In discordant blocks, when demonstrators moved from a minority to a majority choosing the demonstrator optimum, the result was a highly significant increase in the rate at which social learners chose the social learner optimum (Table 2, “Majority demo opt”). In concordant blocks, when demonstrators moved from a minority to a majority choosing the demonstrator optimum, the result was also a highly significant increase in the rate at which social learners chose the social learner optimum (linear combinations, “Majority demo opt + Concordant × Majority demo opt”; Within-subjects, *F* = 28.74, *p* ≪ 0.001; Between-subjects, *F* = 28.77, *p* ≪ 0.001). Concordance had no impact, neither in terms of main effects (Table 2, “Concordant”), in interactions (Table 2, “Concordant × Majority demo opt”), nor as linear combinations of the two (“Concordant + Concordant × Majority demo opt”; Within-subjects, *F* = 0.362, *p* = 0.548; Between-subjects, *F* = 0.958, *p* = 0.328). These results show that the facultative shift in social learning was complete. The key question was whether demonstrators chose their own optimum and not whether learners had the same optimum as demonstrators. When demonstrators did well, social learners also did well, and this was equivalently true regardless of whether demonstrators and social learners had different optima or the same optimum. Simply put, social learners showed no trace of an asymmetric response to social information based on similarity (Fig 2).

**Table 2:** Social learning and optimal choices from experiment 1. Results are from logistic regressions that model social learners choosing the social learner optimum. Independent variables include an index for the 20 learning blocks in each session, a dummy specifying if left was the optimal urn for social learners, a dummy indicating concordance between demonstrators and social learners, a dummy indicating if a majority of demonstrators chose the demonstrator optimum, and the interaction between these last two dummies. Robust standard errors clustered on social learner account for any correlations due to multiple observations per social learner [48, 49], and we show these robust clustered standard errors in parentheses.

Parameter	Within-subjects	Between-subjects
Intercept	-1.946*** (0.570)	-2.780*** (0.737)
Trial block	0.033 (0.023)	0.042 (0.029)
Left optimal (soc)	-0.061 (0.287)	0.751 (0.404)
Concordant	-0.279 (0.531)	-0.143 (0.800)
Majority demo opt	3.897*** (0.673)	4.573*** (0.929)
Concordant × Majority demo opt	0.141 (0.630)	-0.528 (1.200)

*** (0.001)

** (0.01)

* (0.05)

### Discussion

Results from experiment 1 show that our subjects used a completely flexible approach to social learning. Facultative adjustments were complete, and subjects eliminated the stark trade-off, ubiquitous in theoretical studies on the evolution of conformity, between discordance and concordance. They also eliminated the weaker trade-off that would have occurred had they adjusted their use of social information in an asymmetric fashion. Importantly, social cognition in our model can evolve to produce symmetric facultative adjustments like those we observed. This requires the covariance in optima to be sufficiently weak and the signal of similarity to be sufficiently reliable. In our experiment, the covariance was indeed zero, and the signal was perfectly reliable. Our model, however, does not allow learners to evaluate covariance and signal reliability on a case-by-case basis. Covariance and signal reliability are instead inherited representations of the decision-making task. Thus, the symmetric adjustments we observed suggest at least two possibilities.

The first possibility is that weak covariance and reliable signals were characteristic of the evolutionary past, and in our experiment we measured the evolved social learning strategies associated with these past regularities. Importantly, weak covariance and reliable signals may have held in some domains in the past. Consider a sexual division of labor such that women and men had separate roles in society and different optima. A demonstrator was just as likely to be a woman as a man, the covariance in optima would have been approximately zero, and a person’s gender was probably an extremely reliable signal of similarity. Across many domains, however, one can easily imagine that mechanisms like temporal and spatial heterogeneity would have generated positive covariance in optima and moderately informative signals. If so, conditions would have been conducive to the evolution of asymmetric rather than symmetric facultative adjustments. The question then becomes, to what extent are social learning strategies domain-specific? This would be an important topic for future empirical research.

The second possibility is that social learners in our experiment, regardless of the evolutionary past, recognized that learner and demonstrator optima did not covary, signals of similarity were extremely reliable, and symmetric adjustments were thus the best way to make money. This possibility is compelling because the structure of our experimental paradigm was indeed transparent, and so perhaps subjects did not need to rely on evolved social learning strategies. Perhaps all they needed was an intuitive understanding of probability theory and enough mentalizing ability to anticipate that demonstrators would try to identify, with considerable success, the demonstrator optimum. By this reasoning, our experimental results represent the outcome of highly general processes that can interpret frequency-dependent social information but are in no way limited to processing this kind of information.

One might argue that the gene-culture coevolution of cultural transmission, in contrast, specifically concerns heuristics that people apply when the decision-making task is not completely transparent. In such situations, people might turn to evolved social learning strategies as rules of thumb [2, 50] for extracting useful information from the social group. If so, when people are in situations where they do not know what to do but they have access to social information, their choices should reveal intrinsic preferences for specific social learning strategies like following the majority. People may be able to override such preferences if they understand that doing so is in their own material interest, as in our transparent discordant treatments. Otherwise, evolved preferences should reveal themselves. Furthermore, if people use evolved social learning strategies when they cannot tell what to do, these strategies should somehow reflect default assumptions derived from the structure of decision-making at the time the strategy evolved. If concordance, for example, was the typical scenario in the past, social cognition could have evolved, genetically or even culturally [27], to assume concordance in the absence of contradictory information. Again, people may be able to override this default if they have clear information showing that learners and demonstrators are dissimilar, as in our discordant treatments. Otherwise, default assumptions should reveal themselves. To test this reasoning, we conducted a second experiment that examined both preferences for specific social learning strategies and default assumptions about the similarity between social learners and demonstrators.

## Experiment 2

Experiment 2 was similar to the first experiment, with one key exception. We did not inform social learners about their similarity to demonstrators. In some treatments, we withheld this information in a way that maximized the potential for social learners to express specific social learning strategies they might have preferred. In effect, we eliminated any possibility of a conflict between one’s preferred strategy and one’s preference for making money, and so social learners had no reason to do anything but rely on their preferred social learning strategies. In other treatments, we withheld information about similarity in a way that forced social learners to rely on default assumptions about the similarity between social learners and demonstrators. If social cognition, in the absence of contradictory evidence, assumes either discordance or concordance, social learners should have revealed the assumption in these treatments.

### Materials and methods

Each group contained five demonstrators and from five to 11 social learners. The experiment was two-by-two. The first dimension of the experimental design was the same as in experiment 1. Namely, blocks were either discordant or concordant. Unlike experiment 1, however, we did not inform social learners which type of block they faced. Social learners knew the relation with demonstrators in a given block was either discordant or concordant, but they did not know which. For this reason we call these treatments “opaque.” We always implemented the discordant-concordant distinction within social learners with the order counterbalanced across groups within a session.

In the second dimension of the experimental design, we varied whether we told social learners about the prior probabilities of discordant and concordant blocks. In the **prior** treatment, we revealed to social learners that any given block would be either discordant or concordant with equal probability. This feature, combined with the uniform probabilities for assigning balls to urns, ensured that social learners had the information necessary to determine that all social learning strategies were equivalent in terms of expected payoffs. Indeed, they had the information necessary to determine that social learning strategies could not affect the ex ante distribution of payoffs in any way (S4 Appendix). Consequently, if a social learner had some preference for a specific social learning strategy, she could have expressed this preference without concern for the material consequences, and in a open-response survey after the experiment a clear majority of social learners voluntarily expressed an understanding of this (S4 Appendix). Regardless of whether the social learner had preferences over expected payoffs, the variance in payoffs, skew, or kurtosis, all social learning strategies were equivalent in expectation. Because all social learning strategies were equivalent, opaque treatments with known priors removed all countervailing forces that could have prevented a social learner from expressing her preferred social learning strategy.

In the **no prior** treatment, we did not reveal the prior probabilities associated with discordant or concordant blocks, and thus social learners did not have the information necessary to determine that all social learning strategies were ex ante equivalent. Simply by excluding information about the prior, we forced social learners to rely on default assumptions about the similarity between social learners and demonstrators. If social learners tended to assume a uniform prior, the no prior treatment should have been identical to the opaque treatment with the prior. If, however, social learning strategies evolved under conditions in which learners and demonstrators typically had the same optimum, social cognition might revert to this past regularity in the absence of evidence indicating otherwise. If so, the opaque treatment with no prior should have been similar to concordant blocks in experiment 1. Altogether, we conducted six sessions with 55 demonstrators and 87 social learners. Participants received a show-up fee of 10 Swiss Francs, and the average total payment was 36.66 Swiss Francs.

### Results

Demonstrators again identified their own optimal urns effectively. Demonstrators chose their own optima in the final periods of blocks at a rate highly significantly above chance (logistic regression, robust standard errors clustered on demonstrator, *p* ≪ 0.001). Of the five demonstrators in a group, a majority chose the demonstrator optimum in the final period of a block in 95.5% of all blocks. Four out of five choosing optimally was the modal outcome.

**Fig 4:**
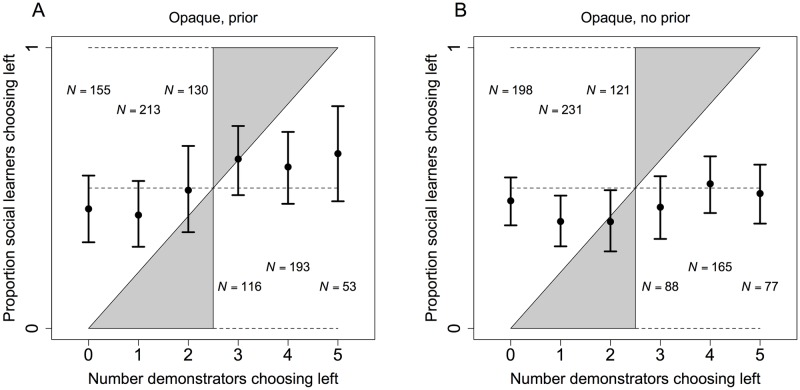
Left choices by social learners in opaque treatments from experiment 2. The points show the rate at which social learners chose left as a function of the number of demonstrators choosing left in the final trials of blocks. The relevant number of observations is vertically aligned with each point. Panel **A** pools over discordant and concordant blocks when social learners knew the prior probabilities associated with discordance versus concordance. Panel **B** shows pooled data when social learners did not know these priors. Error bars are 95% bootstrapped confidence intervals clustered on social learner. Clustering accounts for correlations due to multiple observations per social learner [48], and bootstrapping ensures that confidence intervals remain in [0, 1]. The gray region of the space is consistent with conformist cultural transmission [1, 11]. The diagonal in black is consistent with unbiased social learning, which does not generate cultural evolution, and dashed lines provide additional points of reference at 0, 0.5, and 1.

**Fig 5:**
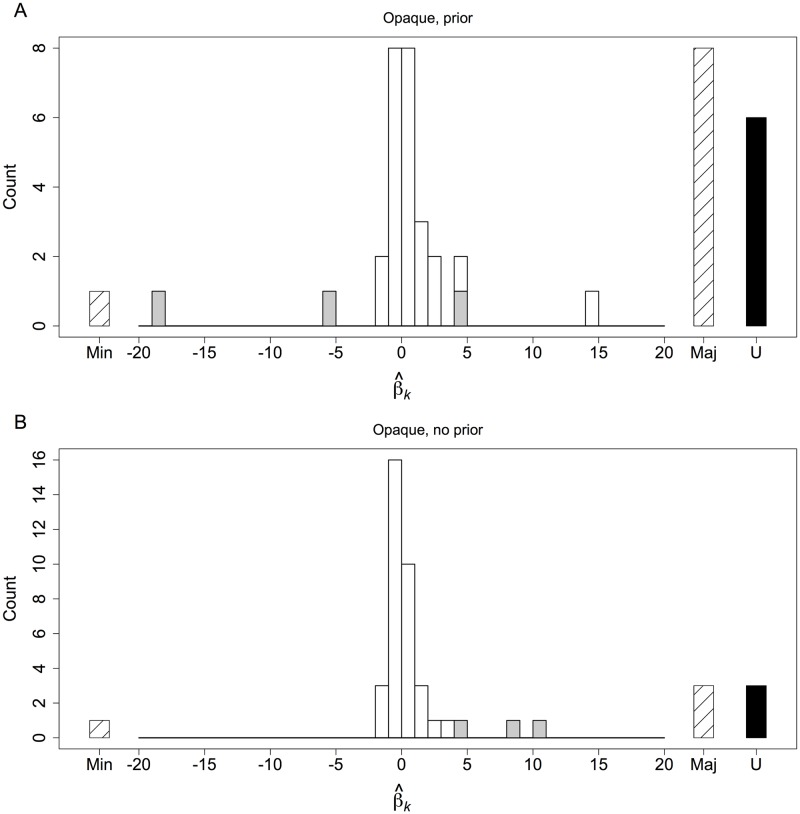
Disaggregated social learnings strategies in opaque treatments from experiment 2. We placed each social learner in one of four categories. A social learner who always followed the minority choice among demonstrators was a “Min” type. One who always followed the majority was a “Maj” type. One who always chose left or always chose right was a “U” type (Unconditional). For the social learners who did not fall into these three categories, we estimated the social learning function of each. Specifically, let *Y*_*jk*_ ∈ {0, 1} indicate if social learner *k* chose left in block *j*, and let *x*_*j*_ be the centered proportion of demonstrators choosing left. We estimated ${\hat{\beta }}_{k}$ by fitting *P*(*Y*_*jk*_ = 1) = (exp{*β*_*k*_ *x*_*j*_})/(1 + exp{*β*_*k*_ *x*_*j*_}) via maximum likelihood. Panels **A** and **B** show the distributions over types for the two opaque treatments with data pooled over discordant and concordant blocks. Gray bars show ${\hat{\beta }}_{k}$ estimates significant at the 5% level. Given multiple tests, we expect two to three significant values by chance in each panel. Social learners who followed the minority (Min) or majority (Maj) and social learners with extreme values of ${\hat{\beta }}_{k}$ (e.g. $|{\hat{\beta }}_{k}|>10$) clearly responded to social information. The rest did not.

For the analyses of social learner choices, we sometimes pool over discordant and concordant blocks (Table 3 and Figs 4 and 5). This approach reflects the fact that social learners could not distinguish between these blocks during the experiment. For other analyses, we condition on whether a block was discordant or concordant (Table 4). Social learners did not have this information, but our fully informed ex post perspective allows us to analyze the data in this way. Doing so, in particular, provides an effective approach to identifying any trade-offs created by a systematic response to social information. For example, following the minority and following the majority were equivalent ex ante. Ex post, however, following the minority would have typically produced large payoffs in discordant but not concordant blocks, while following the majority would have typically produced large payoffs in concordant but not discordant blocks. We test for trade-offs by conditioning on discordance versus concordance in exactly this way (Table 4).

**Table 3:** Social learners choosing left in experiment 2. Results are from logistic regressions for the prior and no prior treatments. Data are pooled over discordant and concordant blocks. Independent variables include an index for the 20 learning blocks in each session and the distribution of choices among demonstrators in the fifth trial of the block. This distribution is centered at zero. Thus, if *p*_5_ is the proportion of demonstrators choosing left in the fifth trial, we transform this as *x*_5_ = *p*_5_ − 0.5. Robust standard errors clustered on social learner account for any correlations due to multiple observations per social learner [48, 49], and we show these robust clustered standard errors in parentheses. The final column combines the analysis of the prior and no prior treatments and shows no significant difference between them. In particular, the estimated rates of choosing left are not significantly different at the extreme values of *x*_5_. Specifically, for the linear combination, “No prior + 0.5 (No prior × Prop left, centered)”, *F* = 2.7684 and *p* = 0.096. For “No prior − 0.5 (No prior × Prop left, centered)”, *F* = 0.005 and *p* = 0.943.

Parameter	Prior	No prior	Combined
Intercept	0.241 (0.191)	-0.076 (0.160)	0.228 (0.175)
Learning block	-0.017 (0.011)	-0.014 (0.012)	-0.0153 (0.008)
Prop left, centered	0.975* (0.476)	0.345 (0.324)	0.975* (0.473)
No prior			-0.291 (0.193)
No prior × Prop left, centered			-0.629 (0.572)

*** (0.001)

** (0.01)

* (0.05)

**Table 4:** Social learning and optimal choices from experiment 2. Results are from logistic regressions that model social learners choosing the social learner optimum. Independent variables include an index for the 20 learning blocks in each session, a dummy specifying if left was the optimal urn for social learners, a dummy indicating concordance between demonstrators and social learners, a dummy indicating if a majority of demonstrators chose the demonstrator optimum, and the interaction between these last two dummies. Robust standard errors clustered on social learner account for any correlations due to multiple observations per social learner [48, 49], and we show these robust clustered standard errors in parentheses.

Parameter	Prior	No prior
Intercept	0.745 (0.468)	0.297 (0.453)
Trial block	0.006 (0.012)	0.003 (0.012)
Left optimal (soc)	0.114 (0.307)	-0.413 (0.228)
Concordant	-1.007 (0.762)	-1.403 (0.780)
Majority demo opt	-1.192* (0.517)	-0.276 (0.521)
Concordant × Majority demo opt	1.623 (0.956)	1.702 (0.919)

*** (0.001)

** (0.01)

* (0.05)

In the prior treatments, the 95% bootstrapped confidence interval clustered on social learner includes 0.5 for any distribution of choices among demonstrators (Fig 4). In this sense, choices were random on average. However, the graph suggests a weakly positive response to social information, and regression results confirm a relatively weak but significantly positive response of this sort (Table 3, Prior, “Prop left, centered”). In treatments with no prior information, aggregate social learning strategies show that the overall response to social information was unsystematic (Fig 4), and regressions lead to the same conclusion. Social learner choices were not significantly related to demonstrator choices (Table 3, No prior, “Prop left, centered”). Importantly, however, an analysis of both the prior and no prior treatments together reveals that social learning strategies did not differ significantly across these two sets of treatments (Table 3, Combined).

With respect to trade-offs, the positive social influence observed in the prior treatments significantly reduced optimal choices among social learners in discordant blocks. Specifically, in discordant blocks the shift from a minority to a majority of demonstrators choosing the demonstrator optimum significantly reduced the rate at which social learners chose the social learner optimum (Table 4, Prior, “Majority demo opt”). In concordant blocks, however, this same shift did not significantly affect the rate at which social learners chose the social learner optimum (Table 4, Prior, linear combination, “Majority demo opt + Concordant × Majority demo opt”, *F* = 0.498, *p* = 0.481). Moreover, social learners in concordant blocks did not choose their optimum significantly more than social learners in discordant blocks, regardless of whether a minority of demonstrators chose the demonstrator optimum (Table 4, Prior, “Concordant”) or a majority (Table 4, Prior, linear combination, “Concordant + Concordant × Majority demo opt”, *F* = 3.460, *p* = 0.0632). All in all, these results show some trade-offs, though limited, in prior treatments. Specifically, for social learners in discordant blocks, the effectiveness of individual learning among demonstrators was a significant distinction. We found no other evidence for trade-offs, however, which is consistent with the observation that the positive social influence observed was relatively weak (Fig 4).

In treatments without the prior, we found no evidence for trade-offs of any kind. The distinction between a minority and a majority of demonstrators choosing the demonstrator optimum had no significant effect on optimal choices among social learners. This result holds when considering the main effect (Table 4, No prior, “Majority demo opt”), the interaction (Table 4, No prior, “Concordant × Majority demo opt”), and the linear combination (Table 4, No prior, linear combination, “Majority demo opt + Concordant × Majority demo opt”, *F* = 2.673, *p* = 0.102). The distinction between discordant and concordant blocks also had no significant effect, neither when a minority of demonstrators chose the demonstrator optimum (Table 4, No prior, “Concordant”) nor when a majority of demonstrators chose the demonstrator optimum (Table 4, No prior, linear combination, “Concordant + Concordant × Majority demo opt”, *F* = 1.560, *p* = 0.212).

The response to social information and associated trade-offs in the prior treatments occurred because a few social learners conformed. To see this, we characterized the social learning strategy of each social learner. We found that the vast majority exhibited no systematic response to social information (Fig 5). 10 of the 43 social learners in the prior treatment, however, responded strongly to social information. Eight of these did so by always following the majority choice among demonstrators, and two exhibited a strong tendency to follow the minority (Fig 5). This imbalance is responsible for the relatively weak but significantly positive response to social information at the aggregate level, a response that also tied the performance of social learners to the performance of demonstrators when social learners were unknowingly in discordant blocks.

In the treatments with no prior, an analysis of the strategy used by each social learner further demonstrates the lack of systematic social learning in these treatments. The vast majority of social learners did not respond to social information (Fig 5). Only four of the 44 social learners responded strongly to social information. Three always followed the majority, and one always followed the minority (Fig 5).

### Discussion

Results from the prior treatments show a tendency to follow the majority among a subset of social learners. These conformists were not especially common, constituting a bit less than a fifth of social learners, but they did consistently follow the majority. Nearly all of the remaining social learners did not respond to social information. Consequently, without a meaningful subset of social learners to offset the conformists, social influence was significantly positive at the aggregate level.

The treatments without priors, in contrast, showed no evidence for strategies that responded systematically to social information. In effect, social learners ignored demonstrators, and they did so even though the distribution of demonstrator choices was the only information they had. This finding suggests that social learners were not willing to make assumptions about their similarity to demonstrators. In particular, in contrast to what we would expect if concordance was typical in the past and continues to shape social learning in the present, they revealed no tendency to assume concordance or to rely on some measure of conformity as a default strategy.

Crucially, however, even though social influence was consistently positive in the prior treatments but not in the no prior treatments, the prior and no prior cases were not significantly different from each other. This suggests that social learners treated the two cases similarly. More to the point, social learners in no prior treatments had no information of any kind about their similarity to demonstrators. They treated this, as suggested by many social learners in an open-response survey after the experiment (S4 Appendix), like a situation in which social learners knew that their optimum and the demonstrator optimum were just as likely to be the same as different. By extension, if behavior in the no prior treatments reflected the degree of similarity in the evolutionary past, genetic or cultural, our result indicates that learner and demonstrator optima were uncorrelated with each other at that time. Another possibility, however, is that social cognition is more complex than this because it allows a more flexible assessment of similarity. For example, in addition to evaluating their similarity to demonstrators, social learners may also be able to evaluate the quality of the information they have about similarity, all as part of a relatively complex and flexible assessment of how to best use frequency-dependent social information. In an experiment with no prior, for example, a uniform prior is arguably a reasonable assumption, whatever the typical covariance between learner and demonstrator optima in the evolutionary past.

## General discussion

What do these findings tell us about the complexity of evolved social cognition and the associated scope for people to modulate their use of frequency-dependent social information according to circumstance? To clarify the issues at hand, we simplify by putting aside individual learning and focusing exclusively on facultative social learning strategies. Switching facultatively between individual and social learning can also be important [43, 51], but we single out questions about adjustments to the use of social information conditional on social information being used.

Consider first a non-facultative social learner. A non-facultative social learner observes the distribution of choices among demonstrators and chooses based on this observation. We will call this a “first-order” strategy because the social learner can respond to variation in demonstrator choices, but she cannot do more. Although most theory on the evolution of frequency-dependent social learning assumes this level of complexity, our results join other recent studies [27, 28] indicating that social learning strategies are more complex than this. The reason is the following. If a social learner can only respond to variation in the distribution of choices, she cannot change how she responds to any specific distribution given the value of some other variable. Social learners, however, actually can do this [27, 28, 52]. We added this kind of complexity in our model by allowing learners to process information about their similarity to the demonstrators from whom they learn. Under appropriate conditions, facultative learning strategies evolve. We will call such strategies “second-order” because a social learner can respond to both information about similarity and variation in the distribution of choices among demonstrators. This is the maximum level of complexity our model supports.

Importantly, however, social learning strategies could be still more complex. For example, aside from responding to both perceived similarity and distributional information, social learners may also be able to evaluate the quality of the information they have about their similarity to demonstrators. This idea would be broadly consistent with our data. To illustrate the logic, social learners in transparent treatments knew they had high quality information about similarity, which led to symmetric switching between following the minority and following the majority. If conditions in the past supported the evolution of asymmetric switching, social learning was nonetheless flexible enough to leave this past regularity aside. Analogously, in opaque treatments, social learners knew they had little or no relevant information about similarity (S4 Appendix), which led most of them to simply ignore demonstrators instead of relying on an inherited preference for conformity or the assumption of concordance. Although our data do not allow us to definitively conclude that this was the relevant cognitive mix, we would like to point out that strategies of this kind would be at least “third-order.”

In any case, our results and others show that facultative responses to frequency-dependent social information can easily outstrip the complexity widely assumed in theoretical work [27, 28]. This finding raises a number of key questions. One key question centers on whether people generally make facultative adjustments based on perceived similarity? Theory shows that arbitrary symbols can evolve to serve as reliable signals of similarity. In particular, initially meaningless symbols can evolve culturally to reflect otherwise unobservable similarities and differences that affect the value of social information [41]. Consequently, reliable markers of similarity are theoretically feasible. Moreover, evaluating similarity seems to be crucial to the development of human social cognition [53], but evaluations are not simply blunt assessments by which a social learner decides whom to imitate and whom to ignore. Rather, infants as young as 18 months have a sophisticated understanding of similarity that allows them to discriminate between observed behaviors that should be imitated, observed behaviors that are superfluous, and observed behaviors that should be avoided [54, 55].

More broadly, young infants demonstrate remarkable sophistication in terms of their ability to infer the preferences and epistemic states of others and to interpret observed behaviors accordingly [56, 57]. This suggests that from a young age social cognition is already equipped to interpret the behavior of others based on situational contingencies. Moreover, a recent fMRI study found that subjects used the opinions of dissimilar others as examples of how not to think [52], a response broadly analogous to following the minority in our transparent discordant treatments. Finally, recent studies of cultural transmission show that people condition their response to social information on circumstance [28, 29, 40, 58, 59], and thus evidence indicates that social learning strategies are often facultative. The number of possible adjustments people can make is potentially huge, and many open questions remain [27].

A second key question centers on the consequences of facultative adjustments. In general, facultative adjustments to the use of social information can have at least two related, fundamental effects. They can alter the cultural evolutionary dynamics that occur, and they can temper the trade-offs between the costs and benefits that would obtain if a non-facultative strategy was applied. Both of these effects will modify natural selection on the social cognition that underpins social learning and cultural transmission. As a result, facultative strategies have the potential to dramatically affect the gene-culture coevolution of human social cognition.

To illustrate, consider the extent to which a conformist bias is like an inflexible heuristic. We can imagine a spectrum of flexibility. At the extreme heuristic end of the spectrum, conformity is like a genetically inherited rigid algorithm with little scope for developmental plasticity. The algorithm takes information about the distribution of behaviors among demonstrators and outputs a behavior without subtlety or consideration of momentary eventualities. Such an algorithm is effective when learners and demonstrators have the same optimum, but it imposes a tremendous drag on optimal choices when learners and demonstrators have different optima. Mesoudi and colleagues [27] describe social learning strategies of this sort as potentially variable across individuals, but fixed at birth for any given individual.

Moving away from rigid first-order algorithms, a social learner may opt for one response to social information or another because of specific triggers during development [27]. Our model is closest to this formulation, but as suggested above the resulting flexibility may still be inadequate in many cases. Moving further along the spectrum, we can imagine that individuals acquire social learning strategies during development via individual or social learning [27]. Interestingly, though this probably occurs for some species [27], such a mechanism cannot explain our data. The social learners in our experiment only received feedback about their performance at the end of experimental sessions, and they never had any information about how other social learners were using social information. Thus, they were never in a position to learn how to learn socially.

Finally, as the most flexible approach to social learning, social learning strategies can arise endogenously from Bayesian inference given a rich set of priors, conditional probabilities, and data. In this case, social learning is at its most flexible precisely because it is not constrained by the structure of the decision-making task in the genetic evolutionary past, the cultural evolutionary past, or even the developmental past. For example, even if a Bayesian’s genetic and cultural ancestors typically faced positive covariance between the optima of learners and demonstrators, and even if the Bayesian herself typically faced positive covariance during her own development, these past regularities need not intrude upon the current strategy of the Bayesian. If the Bayesian knows the current prior probability that she shares an optimum with demonstrators, and if she knows the conditional probabilities necessary to process information about a shared optimum, she can calculate the correct posterior without regard for her genetic, cultural, or developmental heritage. Instead of the past somehow constraining the present, a completely general process integrates all relevant information. Social learning strategies do not exist as independent forms of cognition in this case. They emerge as perfectly flexible responses that follow from ordinary Bayesian inference and the structure of the decision-making task. Evolved and learned strategies can perhaps approximate this kind of flexibility, but they will need to be high-order strategies to do so.

Importantly, evidence suggests that neither rigid heuristics nor Bayesian inferences are likely to typify human social learning. On the one hand, social learning strategies vary from one situation to another [27], and so we know that extreme rigidity is unlikely to hold in many cases. On the other hand, humans sometimes adopt the most common behavior when doing so is obviously sub-optimal [8], and they sometimes conform little when doing so would be optimal [10]. Bayesian maximizers would never make these mistakes. Moreover, in experiments with a combination of individual learning and frequency-dependent social learning, observed dynamics at the aggregate level are about as far as possible from the rational prediction provided by the Bayesian Nash benchmark [60]. This further suggests that strict Bayesian inference is unlikely to be a good description of social cognition. Surprisingly, however, one only has to allow minor deviations from optimal decisions based on Bayesian inference, and theoretical predictions at the aggregate level suddenly match experimental data extremely well [60]. Consequently, if maximizing expected payoffs under Bayesian beliefs does not lead to accurate behavioral predictions, something very close to this benchmark might.

The wrinkle is the following. Even if frequency-dependent social learning is neither a rigid heuristic nor the result of Bayesians maximizing expected payoffs, it can still be closer toward one end of the spectrum or the other. Insofar as social learning strategies like conformity are rigidly applied, they maximize the evolutionary trade-off between the instances when they exploit social information effectively and the instances when they do not. Insofar as social learning strategies are adaptively facultative or effectively learned as the individual develops, strategies can approach the Bayesian ideal, and trade-offs should be less important. Our place between the two extremes is both the result of and the mechanism that shapes the gene-culture coevolution of human social cognition.

A final key question centers on the link between facultative adjustments to the use of social information and the number of behavioral options. In our experiment, as in much of the theory on the evolution of conformity, two behavioral options existed. As a result, following the minority in discordant scenarios was just as good as following the majority in concordant scenarios.

This possibility, however, is unique to situations with two options. If three options exist, eliminating trade-offs is not so easy. Even if social learners know they have a different optimum than demonstrators, this still leaves two options on the table. If social learners know they have the same optimum, however, this focuses attention on a single option. A signal indicating discordance is useful information, but it is less useful than a signal indicating concordance. This discrepancy only becomes larger as the number of possible behaviors increases. Consequently, facultatively switching between individual learning and social learning may become more important, and social learning strategies, when used, may be less flexible. Indeed, recent experimental evidence shows that people rely on conformity more as the number of behavioral options increases [28].

Ultimately, evolved social cognition seems capable of managing, at least to some extent, both facultative switching between individual and social learning and facultative adjustments to how learners respond to social information. The former is a common theme in gene-culture coevolution [26, 34, 43, 51], while the latter is not. Future research should consider how both types of flexibility can influence the evolution of human social cognition and associated cultural evolutionary dynamics.

## Supporting Information

S1 Appendix Gene-culture coevolution when cognition encodes and processes information about similarity. (PDF)

S2 Appendix Asymmetric Bayesian posteriors related to similarity. (PDF)

S3 Appendix Screen shots and excerpts from instructions. (PDF)

S4 Appendix The incentive structure for social learners. (PDF)

S1 Instructions Instructions used in the experiments. (PDF)

S1 Data Raw data from the experiments. (TXT)
