# Supplementary material for: The Evolution of Facultative Conformity Based on Similarity
Source: PLoS One. 2016 Dec 21;11(12):e0168551. doi: 10.1371/journal.pone.0168551 (PMC5176289; doi:10.1371/journal.pone.0168551)
Supplement: S1 Appendix — (PDF) [file pone.0168551.s001.pdf]

S1 Appendix for "The evolution of facultative conformity based on similarity"

Charles Efferson[1,*], Rafael Lalive[2], Maria Paula Cacault[2], Deborah Kistler[2]

1 Department of Economics, University of Zurich, Zurich, Switzerland

2 Department of Economics, University of Lausanne, Lausanne, Switzerland

***charles.efferson@econ.uzh.ch**

Gene-culture coevolution when cognition encodes and processes information about similarity

We begin with the **actual structure of the decision-making task**. We view the situation from the perspective of a naive individual in generation $t+1$ who learns, given both private and social information, and then makes a choice. To designate the environmental state faced by demonstrators in generation t, let Y be a random variable with support $\{0,1\}$ and realizations y. For the state faced by learners in $t+1$, let Z be a random variable with support $\{0,1\}$ and realizations z. The joint prior is $P(y,z)$. Regardless of y, the environmental state changes between t and $t+1$ with probability $\gamma \in [0,1]$. This means that $P(Y=0,Z=0) = P(Y=1,Z=1) = (1-\gamma)/2$, and $P(Y=0,Z=1) = P(Y=1,Z=0) = \gamma/2$. By extension, $P(Y=1) = P(Z=1) = 1/2$, and $\text{cov}(Y,Z) = (1-2\gamma)/4$. As explained below, we will think of this covariance as arising from temporal heterogeneity. More broadly, however, we would like to suggest that this is a secondary consideration. The covariance itself is what matters, however it arises.

Choices are also in $\{0, 1\}$. In any generation, if an individual chooses 0 in state 0 or chooses 1 in state 1, she receives a relatively high payoff, v_H. If she chooses 0 in state 1 or chooses 1 in state 0, she receives a relatively low payoff, v_L, where $0 < v_L < v_H$. Let C_{t+1} be a random variable designating the choice of a randomly selected learner in $t + 1$. C_{t+1} has support $\{0, 1\}$ and realizations c_{t+1}.

Before making a choice, a learner receives three pieces of information. First, the learner receives a private signal about her environmental state, which is a random variable, $\tilde{S}$. The support is some subset of $\mathbb{R}$, and realizations are denoted $\tilde{s}$. We specify how $\tilde{S}$ is distributed below. For the moment, however, we simply note that, because private signals are noisy but informative, $\tilde{S}$ is distributed in a way that depends on the learner's environmental state, and we denote conditional distributions generically as $P(\tilde{S} = \tilde{s} \,|\, Z = z)$. These distributions depend on the state in $t + 1$ but not the state in t, i.e. $P(\tilde{S} = \tilde{s} \,|\, Y = y, Z = z) = P(\tilde{S} = \tilde{s} \,|\, Z = z)$.

Second, the learner in $t + 1$ randomly samples N demonstrators from t and observes how many exhibit behavior 1. This is a random variable, I, with support $\{0, \ldots, N\}$ and realizations i. Let p_t designate the frequency of choice 1 among demonstrators. By extension, $I \sim \text{binomial}(N, p_t)$. Note that, distributions for I depend on the state in t but not the state in $t + 1$, i.e. $P(I = i \,|\, Y = y, Z = z) = P(I = i \,|\, Y = y)$.

Finally, the learner receives a private signal indicating if she is learning in the same environmental state as that of the demonstrators. This signal is a random variable, A, with support $\{0, 1\}$ and realizations a. $A = 1$ indicates the same state. Distributions for A depend on both the state faced by demonstrators and the learner's state. We assume that signals are accurate with probability ϕ, which means
$P(A = 1 \,|\, y = z) = P(A = 0 \,|\, y \neq z) = \phi$.

Now we turn to the **cognitive representation of the decision-making task**. The cognition of learners may or may not accurately represent the actual structure of the task. The point is simply that cognition, whether accurate or not, encodes a representation of the task, and this representation allows learners to choose. We use a hat to indicate quantities that are cognitive representations. This approach will lead to many hats, for which we apologize, but we hope this notation helps to distinguish clearly between the actual structure of the decision-making task and the cognitive representation of this structure.

$\hat{q}_0$ represents the probability that demonstrators choose 1 in state 0, and $\hat{q}_1$ represents the probability that demonstrators choose 1 in state 1. Additionally, learner cognition encodes a prior probability distribution over the four possible (y, z) combinations, where $\hat{w}_{yz}$ represents $P(y, z)$. Cognition also represents the signal quality pertaining to A as $\hat{\phi}$. Given these encoded values, after observing a, i, and $\tilde{s}$, the subjective posterior follows,

$$\hat{P}(y, z \,|\, a, i, \tilde{s}) = \frac{\hat{P}(a \,|\, y, z)\hat{P}(i \,|\, y)\hat{P}(\tilde{s} \,|\, z)\hat{w}_{yz}}{\sum_y \sum_z \hat{P}(a \,|\, y, z)\hat{P}(i \,|\, y)\hat{P}(\tilde{s} \,|\, z)\hat{w}_{yz}}. \tag{1}$$

Let $\hat{E}[V(c_{t+1})]$ be the expected value of choosing c_{t+1} from the learner's perspective. With an exogenous payoff normalized to 1,

$$\begin{aligned}
\hat{E}[V(0)] = 1 &+ \left\{\hat{P}(Y=0, Z=0 \,|\, a, i, \tilde{s}) + \hat{P}(Y=1, Z=0 \,|\, a, i, \tilde{s})\right\} v_H \\
&+ \left\{\hat{P}(Y=0, Z=1 \,|\, a, i, \tilde{s}) + \hat{P}(Y=1, Z=1 \,|\, a, i, \tilde{s})\right\} v_L \\
\hat{E}[V(1)] = 1 &+ \left\{\hat{P}(Y=0, Z=0 \,|\, a, i, \tilde{s}) + \hat{P}(Y=1, Z=0 \,|\, a, i, \tilde{s})\right\} v_L \\
&+ \left\{\hat{P}(Y=0, Z=1 \,|\, a, i, \tilde{s}) + \hat{P}(Y=1, Z=1 \,|\, a, i, \tilde{s})\right\} v_H.
\end{aligned} \tag{2}$$

By substituting (1) into (2) and rearranging, one can show that $\hat{E}[V(1)] > \hat{E}[V(0)]$ if and only if,

$$\begin{aligned}
&\frac{\hat{P}(\tilde{s} \,|\, Z=1)}{\hat{P}(\tilde{s} \,|\, Z=0)} > \\
&\quad \frac{\hat{P}(a \,|\, Y=0, Z=0)\hat{P}(i \,|\, Y=0)\hat{w}_{00} + \hat{P}(a \,|\, Y=1, Z=0)\hat{P}(i \,|\, Y=1)\hat{w}_{10}}{\hat{P}(a \,|\, Y=0, Z=1)\hat{P}(i \,|\, Y=0)\hat{w}_{01} + \hat{P}(a \,|\, Y=1, Z=1)\hat{P}(i \,|\, Y=1)\hat{w}_{11}}.
\end{aligned} \tag{3}$$

Condition (3) is a basic decision-making rule for a learner. If the condition is satisfied, the learner chooses 1. Otherwise, the learner chooses 0. The condition is based on Bayesian updating in the sense that the rule is derived by asking what a Bayesian would do. The condition, however, does not require the learner to do Bayesian updating. Rather, given a cognitive representation of the decision-making task, the learner simply observes a, i, and $\tilde{s}$, checks condition (3), and chooses accordingly.

To clarify that cognition encodes information about the relationship between demonstrators and learners, note that $\hat{r} = \hat{w}_{10} + \hat{w}_{11}$ encodes the prior probability that

$Y = 1$, and $\hat{u} = \hat{w}_{01} + \hat{w}_{11}$ encodes the prior probability that $Z = 1$. Finally, $\hat{D} = \hat{w}_{00}\hat{w}_{11} - \hat{w}_{01}\hat{w}_{10}$ is the encoded value for the covariance between Y and Z. In this sense, $\hat{D}$ captures the encoded prior about the relationship between demonstrators and learners. This representation is modified after observing a, where $\hat{P}(A = 0 \,|\, Y = 0, Z = 0) = \hat{P}(A = 0 \,|\, Y = 1, Z = 1) = 1 - \hat{\phi}$, and $\hat{P}(A = 0 \,|\, Y = 0, Z = 1) = \hat{P}(A = 0 \,|\, Y = 1, Z = 0) = \hat{\phi}$.

Condition (3) comes in two versions, one if $A = 0$ and the other if $A = 1$. Substituting for the $\hat{P}(A = 0 \,|\, y, z)$ and the $\hat{w}_{yz}$ in (3) yields the $A = 0$ condition,

$$\frac{\hat{P}(\tilde{s} \,|\, Z = 1)}{\hat{P}(\tilde{s} \,|\, Z = 0)} > \frac{\hat{P}(i \,|\, Y = 0)(1 - \hat{\phi})((1 - \hat{r})(1 - \hat{u}) + \hat{D}) + \hat{P}(i \,|\, Y = 1)\hat{\phi}(\hat{r}(1 - \hat{u}) - \hat{D})}{\hat{P}(i \,|\, Y = 0)\hat{\phi}((1 - \hat{r})\hat{u} - \hat{D}) + \hat{P}(i \,|\, Y = 1)(1 - \hat{\phi})(\hat{r}\hat{u} + \hat{D})}. \tag{4}$$

Analogously, substituting for the $\hat{P}(A = 1 \,|\, y, z)$ and the $\hat{w}_{yz}$ in (3) yields the $A = 1$ condition,

$$\frac{\hat{P}(\tilde{s} \,|\, Z = 1)}{\hat{P}(\tilde{s} \,|\, Z = 0)} > \frac{\hat{P}(i \,|\, Y = 0)\hat{\phi}((1 - \hat{r})(1 - \hat{u}) + \hat{D}) + \hat{P}(i \,|\, Y = 1)(1 - \hat{\phi})(\hat{r}(1 - \hat{u}) - \hat{D})}{\hat{P}(i \,|\, Y = 0)(1 - \hat{\phi})((1 - \hat{r})\hat{u} - \hat{D}) + \hat{P}(i \,|\, Y = 1)\hat{\phi}(\hat{r}\hat{u} + \hat{D})}. \tag{5}$$

Altogether, the cognition of learning involves six quantities, namely $\hat{r}$, $\hat{u}$, $\hat{D}$, and $\hat{\phi}$, as well as $\hat{q}_0$ and $\hat{q}_1$, which are present in $\hat{P}(i \,|\, Y = 0)$ and $\hat{P}(i \,|\, Y = 1)$ respectively.

To proceed, we **return to the actual structure of the decision-making task.** We introduce simplifying assumptions similar to Perreault and colleagues [1]. Private signals are normally distributed conditional on state. If $Z = 0$, the distribution has mean $\mu_0 < 0$ and variance σ^2. If $Z = 1$, the distribution has mean $\mu_1 = -\mu_0$ and variance σ^2. To normalize private signals, let $\tilde{s} = bs$, where $b = \mu_1$, and thus $s = \tilde{s}/b$ is unit-free. By normalizing in this way, we only need a single quantity to fully specify the stochastic properties of the private signals upon which individual learning depends. Define this single quantity as $\alpha = \sigma^2/(2\mu_1^2)$. To avoid having the clutter of always writing out conditional distributions for normally distributed signals, denote the density for s, given z, as $f_z(s; \alpha)$ and the associated cumulative probability as $F_z(s; \alpha)$.

Turning once again to the cognitive representation of the decision-making task, assume that cognition reflects simplifications analogous to those outlined immediately above. Specifically, let $\hat{\alpha} = \hat{\sigma}^2/(2\hat{\mu}_1^2)$. This means that a learner, in a fashion parallel to reality, views private signals about the environmental state as conditionally normally distributed according $\hat{\alpha}$. In addition, $\hat{r} = \hat{u} = 1/2$ and $\hat{D} = (1 - 2\hat{\gamma})/4$. Finally, $\hat{q}_1 = 1 - \hat{q}_0 = \hat{q}$, which simply means that a learner's cognition encodes the idea that demonstrators make choices that are optimal, from the perspective of the demonstrators, with probability $\hat{q}$. Altogether, $\hat{\alpha}$ summarizes the cognition of individual learning by encoding the information necessary for a learner to interpret a private signal about the environmental state she faces. $\hat{\gamma}$, $\hat{q}$, and $\hat{\phi}$ specify the cognition of social learning in the sense that $\hat{q}$ summarizes the behavior of demonstrators, $\hat{\gamma}$ summarizes the learner's prior regarding the relationship between demonstrators and learners, and $\hat{\phi}$ summarizes how a learner updates her view of this relationship after observing a.

If we denote the cognitive representation of density functions for s given z as $\hat{f}_z(s)$, then the left side of (3) simply becomes $\hat{f}_1(s)/\hat{f}_0(s) = \exp\{s/\hat{\alpha}\}$. Further note that the learner's cognitive representations of observing i take the form,

$$\begin{aligned}\hat{P}(i \,|\, Y = 0) &= \binom{N}{i}(1-\hat{q})^i \hat{q}^{N-i} \\ \hat{P}(i \,|\, Y = 1) &= \binom{N}{i}\hat{q}^i (1-\hat{q})^{N-i}.\end{aligned} \tag{6}$$

By extension, the condition for choosing behavior 1 after observing $A = 0$, i, and s is

$$\exp\left\{\frac{s}{\hat{\alpha}}\right\} > \frac{N(i; \hat{q}, \hat{\gamma}, \hat{\phi}, A = 0)}{B(i; \hat{q}, \hat{\gamma}, \hat{\phi}, A = 0)}, \tag{7}$$

where[1]

$$N(i; \hat{q}, \hat{\gamma}, \hat{\phi}, A = 0) = \hat{q}^{N-i}(1-\hat{q})^i(1-\hat{\phi})(1-\hat{\gamma}) + \hat{q}^i(1-\hat{q})^{N-i}\hat{\phi}\hat{\gamma}$$

$$B(i; \hat{q}, \hat{\gamma}, \hat{\phi}, A = 0) = \hat{q}^{N-i}(1-\hat{q})^i\hat{\phi}\hat{\gamma} + \hat{q}^i(1-\hat{q})^{N-i}(1-\hat{\phi})(1-\hat{\gamma}).$$

[1] We introduce $N(i; \hat{q}, \hat{\gamma}, \hat{\phi}, A = 0)$ and $B(i; \hat{q}, \hat{\gamma}, \hat{\phi}, A = 0)$ because expressions below would otherwise become unruly. To derive, substitute $\hat{r} = \hat{u} = 1/2$, $\hat{D} = (1 - 2\hat{\gamma})/4$, and expression (6) in the right side of (4) and simplify.

Taking natural logarithms and rearranging yields

$$s > \hat{\alpha}\left(\ln(N(i;\hat{q},\hat{\gamma},\hat{\phi},A=0)) - \ln(B(i;\hat{q},\hat{\gamma},\hat{\phi},A=0))\right). \tag{8}$$

Given $A = 0$ and i, which are observed, and z, which is not observed, the probability that the learner chooses behavior 1 is thus

$$\begin{aligned} &P(C_{t+1} = 1 \mid A = 0, i, z) = \\ &\qquad 1 - F_z\left(\hat{\alpha}\left(\ln(N(i;\hat{q},\hat{\gamma},\hat{\phi},A=0)) - \ln(B(i;\hat{q},\hat{\gamma},\hat{\phi},A=0))\right)\right). \end{aligned} \tag{9}$$

Notice that $F_z(\cdot)$ is the actual distribution function for s given z, which is what ultimately matters when specifying the probability that a learner observes a value of s that satisfies condition (8).

Analogously, after observing $A = 1$, i, and s, the condition for choosing behavior 1 is thus

$$\exp\left\{\frac{s}{\hat{\alpha}}\right\} > \frac{N(i;\hat{q},\hat{\gamma},\hat{\phi},A=1)}{B(i;\hat{q},\hat{\gamma},\hat{\phi},A=1)}, \tag{10}$$

where[2]

$$N(i;\hat{q},\hat{\gamma},\hat{\phi},A=1) = \hat{q}^{N-i}(1-\hat{q})^{i}\hat{\phi}(1-\hat{\gamma}) + \hat{q}^{i}(1-\hat{q})^{N-i}(1-\hat{\phi})\hat{\gamma}$$

$$B(i;\hat{q},\hat{\gamma},\hat{\phi},A=1) = \hat{q}^{N-i}(1-\hat{q})^{i}(1-\hat{\phi})\hat{\gamma} + \hat{q}^{i}(1-\hat{q})^{N-i}\hat{\phi}(1-\hat{\gamma}).$$

Taking natural logarithms and rearranging yields

$$s > \hat{\alpha}\left(\ln(N(i;\hat{q},\hat{\gamma},\hat{\phi},A=1)) - \ln(B(i;\hat{q},\hat{\gamma},\hat{\phi},A=1))\right). \tag{11}$$

Given $A = 1$ and i, which are observed, and z, which is not observed, the probability that the learner chooses behavior 1 is

$$\begin{aligned} &P(C_{t+1} = 1 \mid A = 1, i, z) = \\ &\qquad 1 - F_z\left(\hat{\alpha}\left(\ln(N(i;\hat{q},\hat{\gamma},\hat{\phi},A=1)) - \ln(B(i;\hat{q},\hat{\gamma},\hat{\phi},A=1))\right)\right). \end{aligned} \tag{12}$$

To specify how a learning system based on (8) and (11) evolves, we treat cognitive

[2] To derive $N(i;\hat{q},\hat{\gamma},\hat{\phi},A=1)$ and $B(i;\hat{q},\hat{\gamma},\hat{\phi},A=1)$, substitute $\hat{r} = \hat{u} = 1/2$, $\hat{D} = (1-2\hat{\gamma})/4$, and expression (6) in the right side of (5) and simplify.

encodings as genotypes and derive the resulting gene-culture coevolutionary system. Specifically, genotypes are quadruples, $(\hat{\alpha}, \hat{\gamma}, \hat{q}, \hat{\phi})$. Genetically inherited cognitive systems affect choices. Cognitive systems can vary over learners, but the point is that a single learner's cognition allows the learner to process private information about the environment and social information in the form of observed choices among experienced demonstrators. After processing these two forms of information, a learner makes a choice. Because all learners do this, the distribution of observable choices can change through time, a process we can think of as cultural evolution. As the cultural evolutionary process unfolds, the relative ability of different cognitive systems to identify the best choice changes endogenously as a result. This means that the cultural evolutionary process feeds back to change the selective forces controlling the genetic evolution of cognition. In effect, genetically inherited cognitive systems affect cultural evolution, and cultural evolution in turn affects the genetic evolution of cognition.

To see how this works, our task is to derive expressions for both the cultural evolutionary process and the linked genetic evolutionary process. For the cultural evolutionary process, we need expressions for learner choice that depend on neither a, i, or s, all of which vary across learners within a generation[3]. To specify these expressions, recall that p_t designates the actual proportion of demonstrators in t choosing behavior 1. Conditional on z and $z = y$, a learner with genotype $(\hat{\alpha}, \hat{\gamma}, \hat{q}, \hat{\phi})$ chooses 1 in $t+1$ with probability,

$$\begin{aligned}
&P(C_{t+1} = 1 \,|\, z, z = y) \qquad (13)\\
&= P(C_{t+1} = 1 \,|\, z, z = y, A = 0)(1-\phi) + P(C_{t+1} = 1 \,|\, z, z = y, A = 1)\phi \\
&= (1-\phi) \sum_{i=0}^{N} \left\{ 1 - F_z \left(\hat{\alpha} \left(\ln(N(i; \hat{q}, \hat{\gamma}, \hat{\phi}, A = 0)) - \ln(B(i; \hat{q}, \hat{\gamma}, \hat{\phi}, A = 0)) \right) \right) \right\} \binom{N}{i} p_t^i (1-p_t)^{N-i} \\
&\quad + \phi \sum_{i=0}^{N} \left\{ 1 - F_z \left(\hat{\alpha} \left(\ln(N(i; \hat{q}, \hat{\gamma}, \hat{\phi}, A = 1)) - \ln(B(i; \hat{q}, \hat{\gamma}, \hat{\phi}, A = 1)) \right) \right) \right\} \binom{N}{i} p_t^i (1-p_t)^{N-i}.
\end{aligned}$$

Associated cultural evolutionary dynamics are $p_{t+1} = P(C_{t+1} = 1 \,|\, z, z = y)$.

Analogously, conditional on z and $z \neq y$, the learner chooses 1 with probability,

[3] We maintain conditioning on y and z because, as explained below, we actually implemented random changes in the environment when we simulated the system.

$$
\begin{aligned}
&P(C_{t+1} = 1 \,|\, z, z \neq y) \qquad (14)\\
&\quad = P(C_{t+1} = 1 \,|\, z, z \neq y, A = 0)\phi + P(C_{t+1} = 1 \,|\, z, z \neq y, A = 1)(1-\phi)\\
&\quad = \phi \sum_{i=0}^{N} \left\{ 1 - F_z\left(\hat{\alpha}\left(\ln(N(i;\hat{q},\hat{\gamma},\hat{\phi},A=0)) - \ln(B(i;\hat{q},\hat{\gamma},\hat{\phi},A=0))\right)\right)\right\} \binom{N}{i} p_t^i (1-p_t)^{N-i}\\
&\qquad + (1-\phi) \sum_{i=0}^{N} \left\{ 1 - F_z\left(\hat{\alpha}\left(\ln(N(i;\hat{q},\hat{\gamma},\hat{\phi},A=1)) - \ln(B(i;\hat{q},\hat{\gamma},\hat{\phi},A=1))\right)\right)\right\} \binom{N}{i} p_t^i (1-p_t)^{N-i}.
\end{aligned}
$$

Associated cultural evolutionary dynamics are $p_{t+1} = P(C_{t+1} = 1 \,|\, z, z \neq y)$. Notice that cultural evolutionary dynamics are conditioned on (y, z).

To simulate the gene-culture coevolutionary system numerically, we represented the genotype space on a lattice. Specifically, we created a set $\mathcal{A}$ consisting of 51 evenly spaced $\hat{\alpha}$ values from 0.01 to 20.01. For $\hat{\gamma}$ we created the set $\mathcal{G}$ consisting of 11 evenly spaced values from 0.0001 to 0.9999. Similarly, for $\hat{q}$ we created $\mathcal{Q}$ consisting of 11 evenly spaced values from 0.0001 to 0.9999, and for $\hat{\phi}$ we created $\mathcal{P}$ consisting of 11 evenly spaced values from 0.0001 to 0.9999. The genotype space was $\mathcal{A} \times \mathcal{G} \times \mathcal{Q} \times \mathcal{P}$.

Given the resulting $J = 67,881$ genotypes, we index genotypes by j, $\hat{h}_j = (\hat{\alpha}_j, \hat{\gamma}_j, \hat{q}_j, \hat{\phi}_j)$. $\mathbf{\Theta}_t = [\theta_{1,t}\ \theta_{2,t}\ \ldots\ \theta_{J,t}]^\top$ is the distribution over genotypes in t, where, $\forall j \in \{1, \ldots, J\}$, $\theta_{j,t} \geq 0$ and $\sum_j \theta_{j,t} = 1$. We set $v_L = 0$ and $v_H = 1$. The expected fitness of $\hat{h}_j$ is thus

$$
\begin{aligned}
V_t(\hat{h}_j) = 1 &+ (1-y)(1-z)(1 - P(C_t = 1 \,|\, Y = 0, Z = 0, \hat{h}_j))\\
&+ (1-y)zP(C_t = 1 \,|\, Y = 0, Z = 1, \hat{h}_j)\\
&+ y(1-z)(1 - P(C_t = 1 \,|\, Y = 1, Z = 0, \hat{h}_j))\\
&+ yzP(C_t = 1 \,|\, Y = 1, Z = 1, \hat{h}_j).
\end{aligned}
$$

$\boldsymbol{V}_t = [V_t(\hat{h}_1)\ V_t(\hat{h}_2)\ \ldots\ V_t(\hat{h}_J)]^\top$ is a vector of expected fitness values. Genetic evolutionary dynamics are, $\forall j$,

$$\theta_{j,t+1} = \theta_{j,t} V_t(\hat{h}_j) / (\mathbf{\Theta}_t^\top \boldsymbol{V}_t). \qquad (15)$$

Expressions (13), (14), and (15) specify the gene-culture coevolutionary system.

We simulated 1,000,000 generations. Simulations changed environmental states exogenously with probability γ between adjacent generations. For all simulations, $N = 5$. To specify the remaining parameter combinations, we considered $\gamma \in \{0.01, 0.1\}$, $\phi \in \{0.5, 0.7, 0.9, 0.95\}$, and, given $\mu_1 = 1$, $\sigma \in \{1, 5, 9\}$. We later considered additional parameter combinations by implementing simulations based on $\gamma \in \{0.25, 0.5\}$, $\phi \in \{0.7, 0.9\}$, and $\sigma = 9$.

We would like to emphasize four results. First, when the signal of similarity is not reliable ($\phi = 0.5$), facultative strategies that condition on a do not evolve (Figs A and B). Second, when the signal of similarity is informative (e.g. $\phi = 0.9$), facultative strategies evolve when individual learning is relatively effective. In this case, individual learning has a relatively strong influence when the signal of similarity indicates discordance (Figs A and B), and the learning system exhibits strongly positive social influence with the "S" shape of conformity when the signal of similarity indicates concordance (Figs A and B). Third, when the signal of similarity is informative (e.g. $\phi = 0.9$), facultative strategies may or may not evolve when individual learning is relatively ineffective. If the probability of discordance is low (e.g. $\gamma = 0.01$), strategies are not meaningfully facultative, and they exhibit positive social influence with the "S" shape of conformity (Fig A). If the probability of discordance is higher (e.g. $\gamma = 0.1$), strategies are strongly facultative. They exhibit negative social influence when the signal of similarity indicates discordance (Fig B) and positive social influence when the signal indicates concordance (Fig B). Adjustments in this case are asymmetric, but relatively reliable signals and relatively high probabilities of discordance reduce the asymmetry (S2 Appendix).

Finally, steady-state learning is polymorphic in terms of cognition but not in terms of phenotype. Specifically, for all parameter combinations, two or more cognitive representations are present in equilibrium. They are essentially indistinguishable phenotypically, but they represent the structure of the decision-making task in very different ways. To see the intuition, imagine that copying the majority behavior among demonstrators is advantageous. Cognition can produce conformity as a behavioral response in at least two different ways. It can encode the idea that demonstrators are biased toward the demonstrator optimum, and demonstrators and learners have the same optimum. Alternatively, it can encode the idea that demonstrators are biased

toward the demonstrator sub-optimum, and demonstrators and learners have different optima. Importantly, within the context of our model, these two encodings can be behaviorally equivalent. They cannot, however, both be accurate representations of reality. If selection favors accurate representations in other decision-making domains, this might eliminate one or more representations in the domain we consider. In any case, selection ultimately responds to phenotype, and phenotypes are equivalent.

Table A. Summary of quantities in the gene-culture model given by (13), (14), and (15).

Random variables

Random variable	Realization	Support	Description
Y	y	$\{0,1\}$	Demonstrator state
Z	z	$\{0,1\}$	Learner state
C_{t+1}	c_{t+1}	$\{0,1\}$	Learner choice
A	a	$\{0,1\}$	Signal indicating similarity between learner and demonstrators (social learning)
I	i	$\{0,\dots,N\}$	Sampled demonstrators choosing 1 (social learning)
S	s	$\mathbb{R}$	Normalized private signal (individual learning)

Actual structure of decision-making task

Parameter or function	Description
α	Unit-free dispersion of S, i.e. reliability of individual learning
γ	Probability that learner and demonstrators have different optima
ϕ	Probability that a correctly indicates whether learner and demonstrators have the same optimum
N	Number of demonstrators sampled by learner
$F_0(\cdot)$	Cumulative distribution for S given $Z=0$, $E[S \mid Z=0]=-1$
$F_1(\cdot)$	Cumulative distribution for S given $Z=1$, $E[S \mid Z=1]=1$

Cognitive representation of decision-making task (i.e. genotype)

Variable	Description
$\hat{\alpha}$	Unit-free dispersion of S, i.e. reliability of individual learning
$\hat{\gamma}$	Probability that learner and demonstrators have different optima
$\hat{q}$	Probability that demonstrators choose their optimum
$\hat{\phi}$	Probability that a correctly indicates whether learner and demonstrators have the same optimum

State variables (i.e. gene-culture coevolution)

Variable	Description
p_t	Proportion of decision makers choosing 1 in t ($p_{t+1} \neq p_t \Rightarrow$ cultural evolution)
$\mathbf{\Theta}_t = [\theta_{1,t}\ \theta_{2,t}\ \dots\ \theta_{J,t}]^\top$	Distribution over genotypes in t ($\mathbf{\Theta}_{t+1} \neq \mathbf{\Theta}_t \Rightarrow$ genetic evolution)

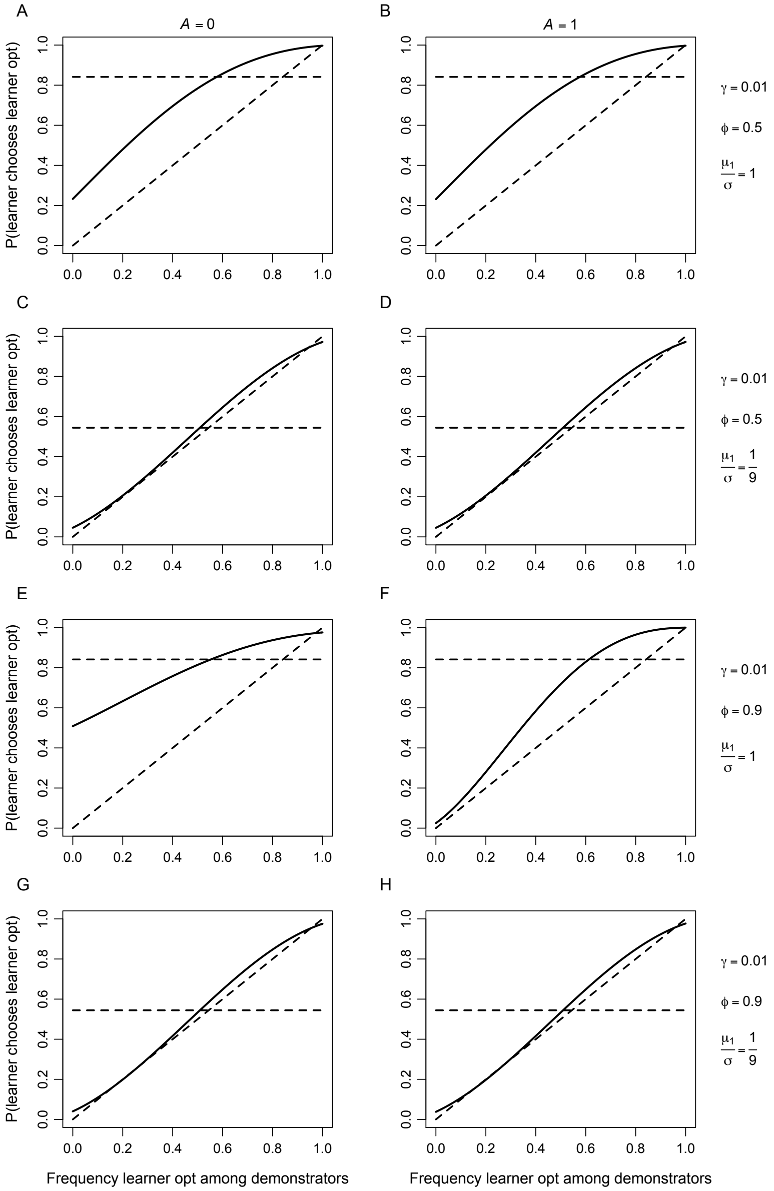

Figure A. Evolved learning strategies when learners and demonstrators have the same optimum with a relatively high probability. Solid lines summarize the properties of the learning system by showing the probability that learners choose their own optimum as a function of how common this same behavior is among demonstrators. Learning strategies potentially depend on whether a learner receives a signal indicating either different optima ($A = 0$) or the same optimum ($A = 1$) for learners and demonstrators. Demonstrators and learners have the same optimum with relatively high probability ($1 - \gamma = 0.99$). Rows vary according to whether the signal indicating similarity is uninformative ($\phi = 0.5$) or informative ($\phi = 0.9$) and whether individual learning is relatively accurate ($\mu_1/\sigma = 1$) or inaccurate ($\mu_1/\sigma = 1/9$). The horizontal dashed lines show a learning system that ignores demonstrator behavior and relies only on individual learning. The diagonal dashed lines show an unbiased learning system that does not generate cultural evolution.

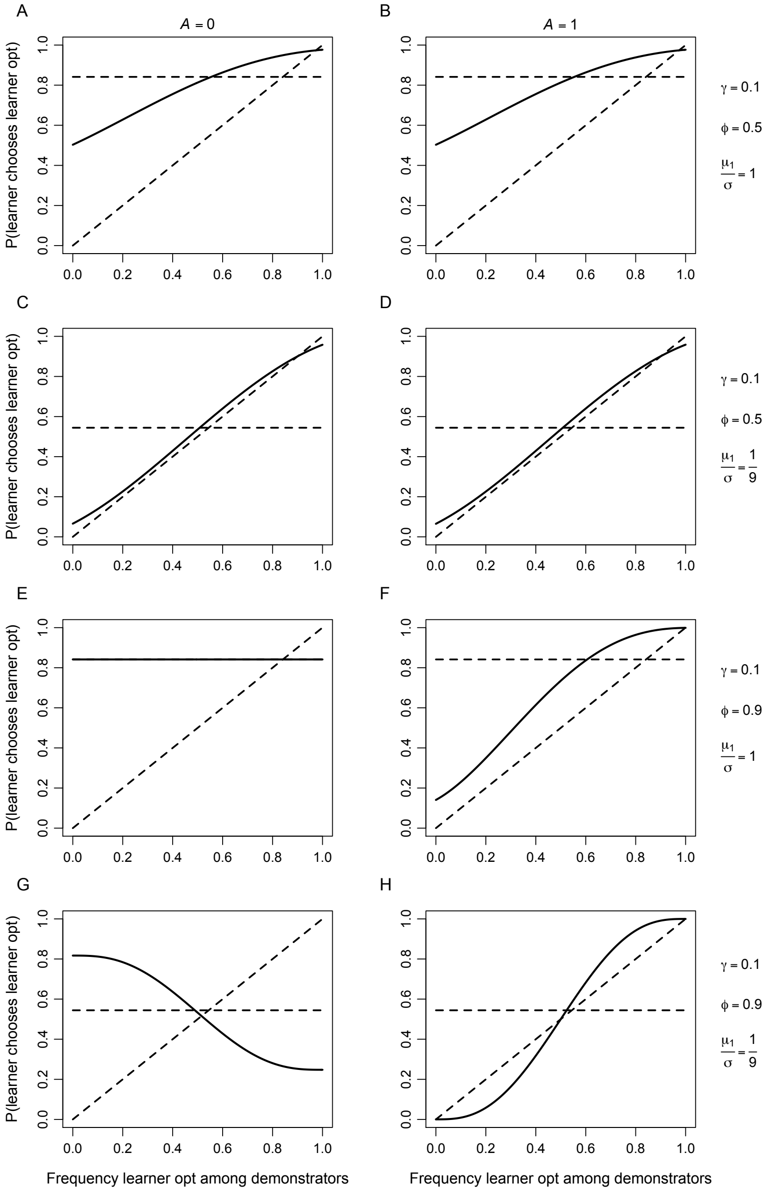

Figure B. Evolved learning strategies when learners and demonstrators have the same optimum with an intermediate probability. Solid lines summarize the properties of the learning system by showing the probability that learners choose their own optimum as a function of how common this same behavior is among demonstrators. Learning strategies potentially depend on whether a learner receives a signal indicating either different optima ($A = 0$) or the same optimum ($A = 1$) for learners and demonstrators. Demonstrators and learners have the same optimum with an intermediate probability ($1 - \gamma = 0.9$). Rows vary according to whether the signal indicating similarity is uninformative ($\phi = 0.5$) or informative ($\phi = 0.9$) and whether individual learning is relatively accurate ($\mu_1/\sigma = 1$) or inaccurate ($\mu_1/\sigma = 1/9$). The horizontal dashed lines show a learning system that ignores demonstrator behavior and relies only on individual learning. The diagonal dashed lines show an unbiased learning system that does not generate cultural evolution.

References

1. Perreault C, Moya C, Boyd R. A Bayesian approach to the evolution of social learning. Evolution and Human Behavior. 2012;33(5):449 – 459. Available from: http://www.sciencedirect.com/science/article/pii/S1090513811001437.
