# Supplementary material for: The Evolution of Facultative Conformity Based on Similarity
Source: PLoS One. 2016 Dec 21;11(12):e0168551. doi: 10.1371/journal.pone.0168551 (PMC5176289; doi:10.1371/journal.pone.0168551)
Supplement: S2 Appendix — (PDF) [file pone.0168551.s002.pdf]

S2 Appendix for "The evolution of facultative conformity based on similarity"

Charles Efferson[1,*], Rafael Lalive[2], Maria Paula Cacault[2], Deborah Kistler[2]

1 Department of Economics, University of Zurich, Zurich, Switzerland

2 Department of Economics, University of Lausanne, Lausanne, Switzerland

***charles.efferson@econ.uzh.ch**

Asymmetric Bayesian posteriors related to similarity

Let $P(Z = y) \in (0.5, 1)$, which indicates that the prior probability for similarity is biased in favor of a shared optimum. Further let $P(A = 0 \,|\, Z \neq y) = \phi$ and $P(A = 1 \,|\, Z = y) = \phi$, where $\phi \in (0.5, 1)$. Because $P(A = 0 \,|\, Z \neq y) = P(A = 1 \,|\, Z = y)$, the signal of similarity is symmetric. Because $\phi \in (0.5, 1)$, the signal is noisy but informative. The posterior probabilities for a shared optimum are

$$P(Z = y \,|\, A = 1) = \frac{\phi P(Z = y)}{(1-\phi)(1 - P(Z = y)) + \phi P(Z = y)}$$

$$P(Z \neq y \,|\, A = 0) = \frac{\phi(1 - P(Z = y))}{\phi(1 - P(Z = y)) + (1-\phi)P(Z = y)}.$$

By extension,

$$P(Z = y \,|\, A = 1) - P(Z \neq y \,|\, A = 0) = \frac{\phi(1-\phi)(2P(Z = y) - 1)}{P(Z = y)(1 - P(Z = y)) + \phi(1-\phi)(2P(Z = y) - 1)^2}. \tag{16}$$

Given our assumptions, the difference in (16) is necessarily positive. Equivalently, the posterior after observing a signal consistent with a biased prior (i.e. $A = 1$) has less

entropy than the posterior after observing a signal opposed to the biased prior (i.e. $A = 0$). This asymmetry is at the root of the asymmetric adjustments that evolve in the model presented in S1 Appendix when $\gamma < 0.5$. Notice, however, that we can make the asymmetry in (16) arbitrarily small by letting $P(Z = y) \to 0.5^{+}$ or $\phi \to 1^{-}$. Evolutionary results also show that the asymmetry associated with facultative adjustments depends jointly on $P(Z = y) = \gamma$ and ϕ (Fig C).

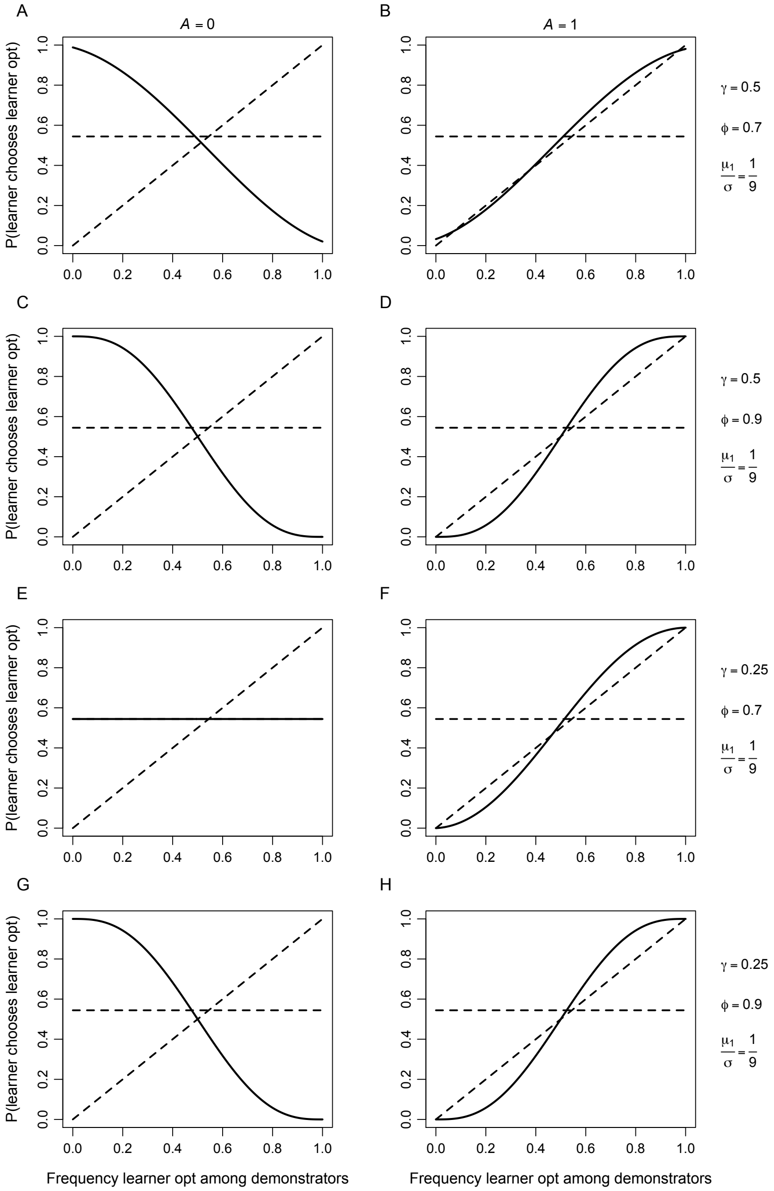

Figure C. The probability of discordance and the reliability of the signal of similarity jointly affect the symmetry or asymmetry of facultative adjustments. Solid lines show the probability that learners choose their own optimum as a function of how common this same behavior is among demonstrators. Learning strategies potentially depend on whether a learner receives a signal indicating either different optima ($A = 0$) or the same optimum ($A = 1$) for learners and demonstrators. Rows vary according to whether the signal indicating similarity is somewhat informative ($\phi = 0.7$) or more informative ($\phi = 0.9$) and whether demonstrators and learners have the same optimum with a probability of either $1 - \gamma = 0.5$ or $1 - \gamma = 0.75$. Individual learning is relatively inaccurate ($\mu_1/\sigma = 1/9$). The horizontal dashed lines show a learning system that ignores demonstrator behavior and relies only on individual learning. The diagonal dashed lines show an unbiased learning system that does not generate cultural evolution. Results show that, when the optima of learners and demonstrators do not covary ($1 - \gamma = 0.5$), informative signals ($\phi > 0.5$) support the evolution of facultative adjustments that are symmetric. When optima covary positively ($1 - \gamma > 0.5$), evolved facultative adjustments are asymmetric, but the asymmetry diminishes as the signal of similarity becomes more reliable (e.g. ϕ increasing from 0.7 to 0.9).
