# Supplementary material for: The Evolution of Facultative Conformity Based on Similarity
Source: PLoS One. 2016 Dec 21;11(12):e0168551. doi: 10.1371/journal.pone.0168551 (PMC5176289; doi:10.1371/journal.pone.0168551)
Supplement: S3 Appendix — (PDF) [file pone.0168551.s003.pdf]

S3 Appendix for "The evolution of facultative conformity based on similarity"

Charles Efferson[1,*], Rafael Lalive[2], Maria Paula Cacault[2], Deborah Kistler[2]

1 Department of Economics, University of Zurich, Zurich, Switzerland

2 Department of Economics, University of Lausanne, Lausanne, Switzerland

***charles.efferson@econ.uzh.ch**

Screens shots and excerpts from instructions

Immediately below we include a selection of screen shots from the experiment (Figs D - J). These show decision and feedback screens for both demonstrators and social learners, and they show the screens that distinguish transparent and opaque treatments. In particular, the caption to Fig H provides excerpts from the instructions to show the features distinguishing the two opaque treatments from each other. The full instructions for all treatments, translated from French, are also available as S1 Instructions.

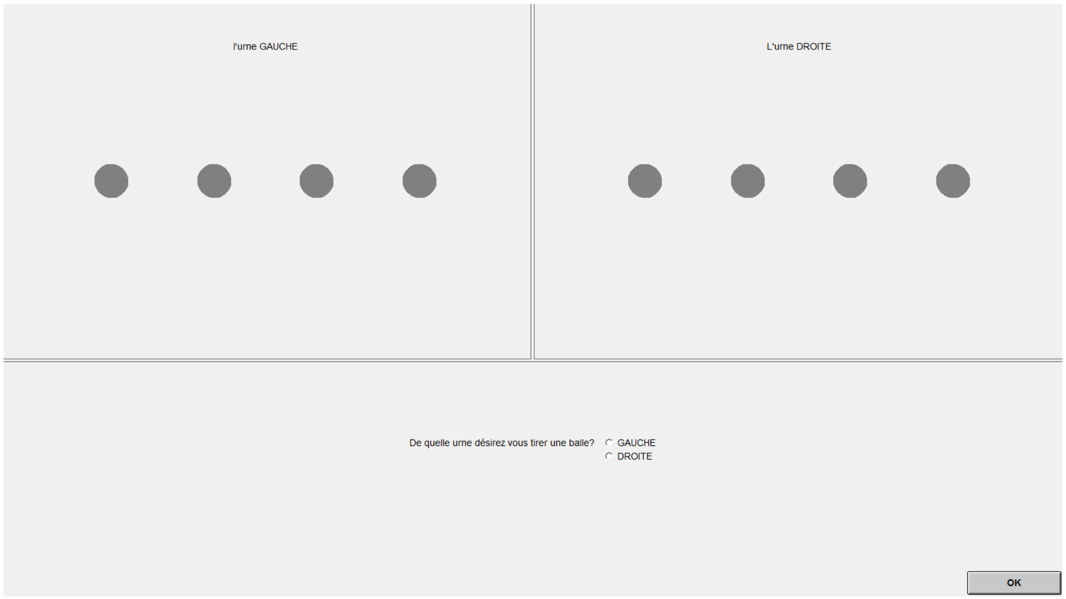

Figure D. Choice screen for demonstrators. Demonstrators faced two urns, one on the left (“GAUCHE”) and one on the right (“DROITE”). One urn contained three red balls and one blue ball, while the other urn contained one red ball and three blue balls. In each period, a demonstrator chose an urn. Choice options were aligned vertically, and the option on top was randomized for each subject and each trial. A single ball was sampled from the chosen urn and its color revealed (Fig E). Demonstrators received 100 points for a ball of one color but nothing for a ball of the other color. The two sets of balls were randomly allocated to the urns, which determined which urn was optimal, at the beginning of each block of five periods. Sessions consisted of 20 blocks of five periods each. Points were converted to cash at the end of a session.

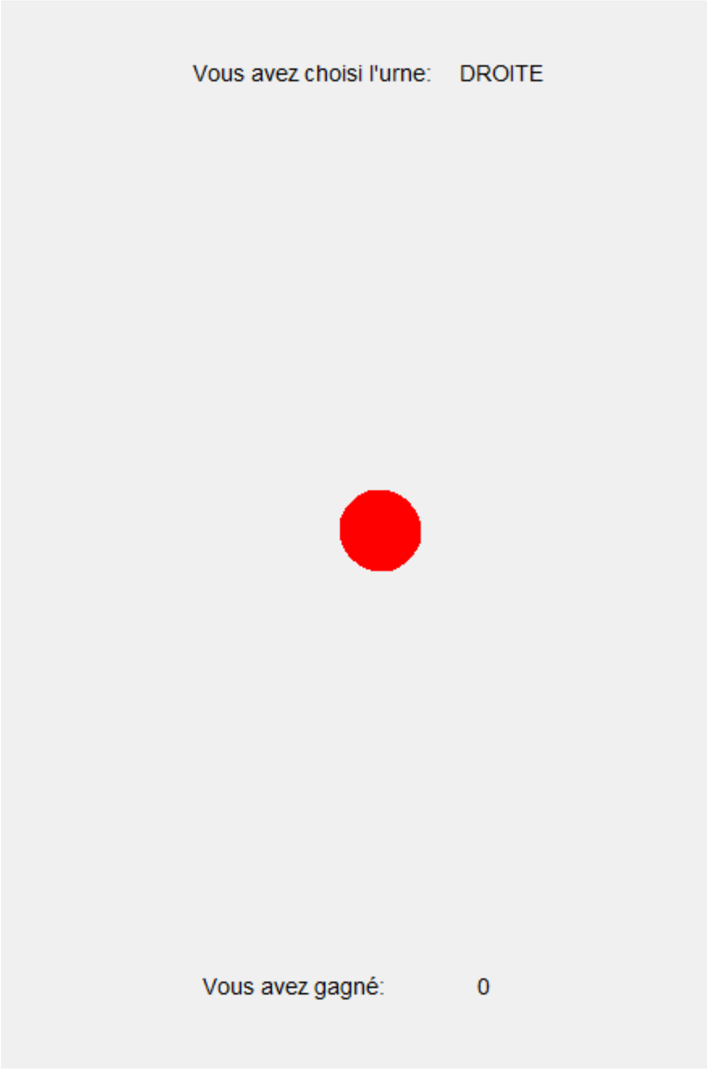

Figure E. An example payoff screen for a demonstrator. In this example, the demonstrator chose the urn on the right ("DROITE"), and a red ball was randomly drawn from this urn. In this case a red ball produced 0 points.

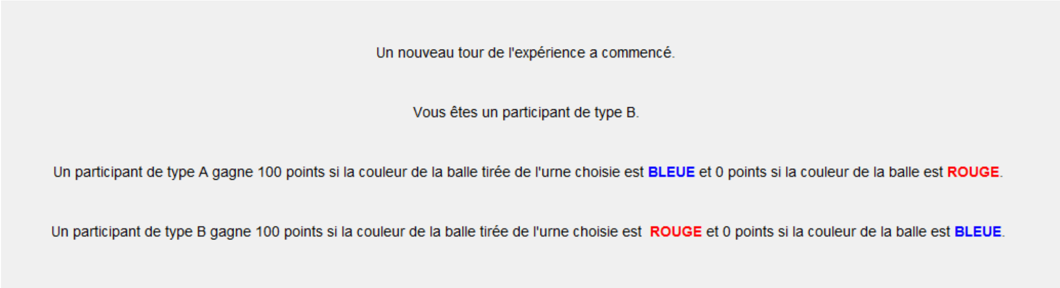

Figure F. An example screen identifying the beginning of a transparent discordant block. All participants saw a screen of this type at the beginning of each block of five periods, but in this example the screen is specifically for a social learner ("un participant de type B"). The third line explains that a demonstrator ("[u]n participant de type A") earns 100 points for a blue ball sampled from the chosen urn and no points for a red ball sampled from the chosen urn. The fourth line explains that a social learner earns 100 points for a red ball sampled from the chosen urn and no points for a blue ball sampled from the chosen urn.

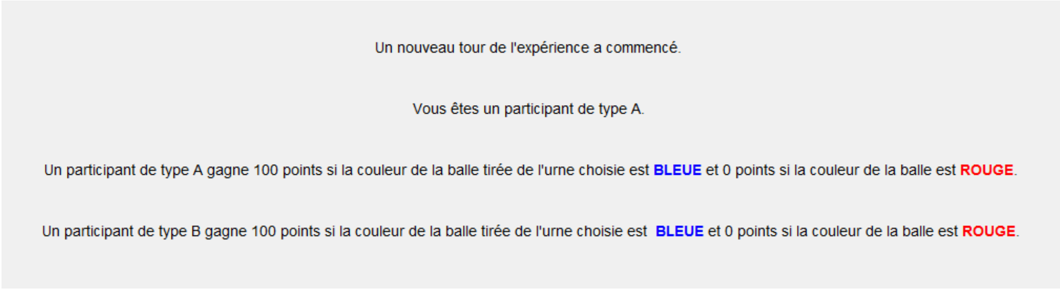

Figure G. An example screen identifying the beginning of a transparent concordant block. All participants saw a screen of this type at the beginning of each block of five periods, but in this example the screen is specifically for a demonstrator ("un participant de type A"). The third line explains that a demonstrator earns 100 points for a blue ball sampled from the chosen urn and no points for a red ball sampled from the chosen urn. The fourth line explains that a social learner ("[u]n participant de type B") earns 100 points for a blue ball and no points for a red ball.

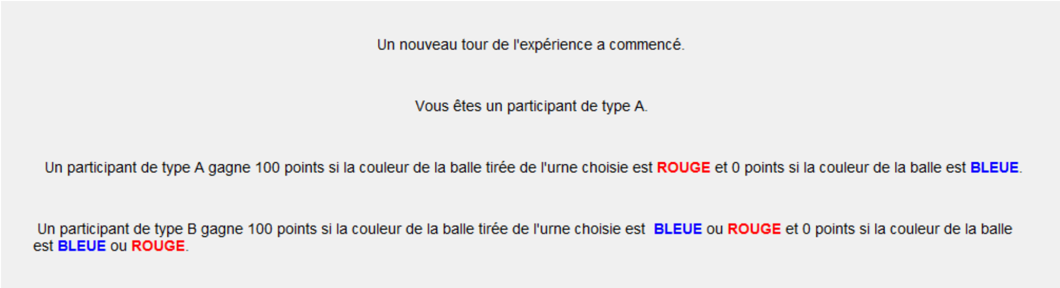

Figure H. An example screen identifying the beginning of an opaque block. All participants saw a screen of this type at the beginning of each block of five periods, but in this example the screen is specifically for a demonstrator ("un participant de type A"). The third line explains that a demonstrator earns 100 points for a red ball sampled from the chosen urn and no points for a blue ball sampled from the chosen urn. The fourth line is completely uninformative by design and explains that a social learner ("[u]n participant de type B") earns 100 points for a blue or red ball and no points for a blue or red ball. The printed instructions, which were available to participants for the duration of a session, explained this statement further. For the opaque sessions with priors, these instructions read, "La couleur gagnante des participants de type B peut être, soit ROUGE, soit BLEUE, avec la même probabilité. Si la couleur gagnante est ROUGE, ils gagnent 100 points si la balle tirée de l'urne choisie est rouge, tandis qu'ils gagnent 0 point si la balle tirée de l'urne choisie est bleue. Au contraire, si la couleur gagnante est BLEUE, ils gagnent 0 point si la balle tirée de l'urne choisie est rouge, tandis qu'ils gagnent 100 points si la balle tirée de l'urne choisie est bleue." This translates as, "The winning color for participants of type B can be either RED or BLUE, with the same probability. If the willing color is RED, they win 100 points if the ball drawn from the chosen urn is red and 0 points if the ball drawn from the chosen urn is blue. In contrast, if the winning color is BLUE, they win 0 points if the ball drawn from the chosen urn is red and 100 points if the ball drawn from the chosen urn is blue." For the opaque sessions without priors, the analogous section of the instructions read, "Ils gagnent 100 points si la balle tirée de l'urne choisie est de la couleur gagnante, et ils gagnent 0 point si la balle tirée de l'urne choisie n'est pas de la couleur gagnante." Translated, "They win 100 points if the ball drawn from the chosen urn is the winning color, and they win 0 points if the ball drawn from the chosen urn is not the winning color."

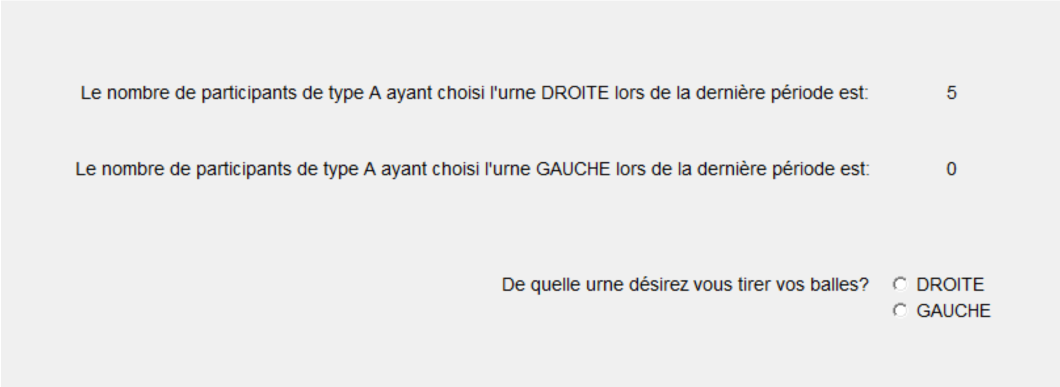

Figure I. An example choice screen for social learners. The screen reports the number of demonstrators choosing the right urn ("l'urne DROITE") and the left urn ("l'urne GAUCHE") in the last period of the block ("la dernière période"). It then asks the social learner to choose an urn. Choice options were aligned vertically, and the option on top was randomized for each subject and each trial.

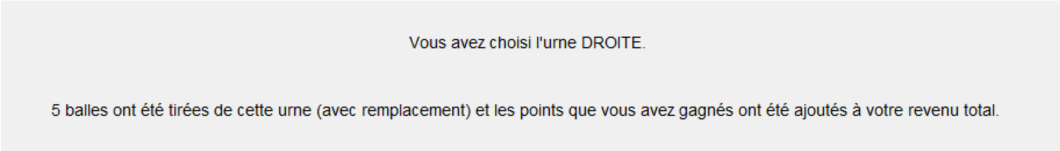

Figure J. An example feedback screen for social learners. The screen reports that the social learner in question chose the right urn ("l'urne DROITE"). It further states that five balls were drawn with replacement from this urn, and any points earned have been added to the social learner's total revenue.
