# Supplementary material for: The Evolution of Facultative Conformity Based on Similarity
Source: PLoS One. 2016 Dec 21;11(12):e0168551. doi: 10.1371/journal.pone.0168551 (PMC5176289; doi:10.1371/journal.pone.0168551)
Supplement: S4 Appendix — (PDF) [file pone.0168551.s004.pdf]

S4 Appendix for "The evolution of facultative conformity based on similarity"

Charles Efferson[1,*], Rafael Lalive[2], Maria Paula Cacault[2], Deborah Kistler[2]

1 Department of Economics, University of Zurich, Zurich, Switzerland

2 Department of Economics, University of Lausanne, Lausanne, Switzerland

***charles.efferson@econ.uzh.ch**

The incentive structure for social learners

Let payoffs for a social learner be a random variable, Π, with support $\{0, \bar{\pi}\}$ and realizations π. In our experiment, $\bar{\pi} = 100$. Denote the the probability of receiving $\bar{\pi}$ if choosing sub-optimally as ψ_L. The probability of receiving $\bar{\pi}$ if choosing optimally is ψ_H. In our experiment, $\psi_L = 1/4$, and $\psi_H = 3/4$. Let $f : \{0, \ldots, N\} \rightarrow \{0, 1\}$ be an arbitrary social learning function that specifies a behavior for the social learner as a function of i, the number of demonstrators choosing 1 (e.g. left). $N = 5$ in our experiment, and we treat N as odd below to ensure that a clear majority choice among demonstrators always exists. We retain the remaining notation from S1 Appendix.

We now examine the incentives faced by social learners in the different treatments. In particular, we show that, if demonstrators actually learn, all central moments associated with the payoff distribution are independent of f in an opaque setting with priors available. This means that, if a social learner has any preference for adhering to a specific social learning strategy, she can express this preference without cost. Whatever her preference, it has no material consequences in terms of the expected payoff, the variance in payoffs, skew, or kurtosis.

The k-th moment about the origin takes the form,

$$
\begin{aligned}
E[\Pi^k \mid f] = \hat{w}_{00} \sum_i \binom{N}{i} \hat{q}_0^i (1-\hat{q}_0)^{N-i} \left\{ f(i)\bar{\pi}^k \psi_L + (1-f(i))\bar{\pi}^k \psi_H \right\} \\
+ \hat{w}_{01} \sum_i \binom{N}{i} \hat{q}_0^i (1-\hat{q}_0)^{N-i} \left\{ f(i)\bar{\pi}^k \psi_H + (1-f(i))\bar{\pi}^k \psi_L \right\} \\
+ \hat{w}_{10} \sum_i \binom{N}{i} \hat{q}_1^i (1-\hat{q}_1)^{N-i} \left\{ f(i)\bar{\pi}^k \psi_L + (1-f(i))\bar{\pi}^k \psi_H \right\} \\
+ \hat{w}_{11} \sum_i \binom{N}{i} \hat{q}_1^i (1-\hat{q}_1)^{N-i} \left\{ f(i)\bar{\pi}^k \psi_H + (1-f(i))\bar{\pi}^k \psi_L \right\}.
\end{aligned}
$$

Assume that demonstrators actually learn and are equally like to choose their own optimum regardless of whether it is left or right. This implies $\hat{q}_1 = 1 - \hat{q}_0 = \hat{q} > 1/2$. Letting $\hat{c} = 2\hat{u} - 1$, the k-th moment becomes the following,

$$
\begin{aligned}
E[\Pi^k \mid f] = \pi^k \Bigg[\hat{u}\psi_L + (1-\hat{u})\psi_H \\
+ (\psi_H - \psi_L) \sum_{i=0}^{\lfloor N/2 \rfloor} \binom{N}{i} (1-\hat{q})^i \hat{q}^{N-i} f(i) \left\{ \hat{c} - (\hat{r}c + 2\hat{D}) \left(1 - \left(\frac{1-\hat{q}}{\hat{q}} \right)^{N-2i} \right) \right\} \\
+ (\psi_H - \psi_L) \sum_{i=\lceil N/2 \rceil}^{N} \binom{N}{i} \hat{q}^i (1-\hat{q})^{N-i} f(i) \left\{ \hat{c} \left(\frac{1-\hat{q}}{\hat{q}} \right)^{2i-N} + (\hat{r}c + 2\hat{D}) \left(1 - \left(\frac{1-\hat{q}}{\hat{q}} \right)^{2i-N} \right) \right\} \Bigg].
\end{aligned}
$$

One can easily verify that, if $\hat{u} = 1/2$ and $\hat{D} < 0$, which would correspond to information explicitly provided in transparent discordant treatments, the social learner maximizes the expected payoff by perfectly following the minority. Similarly, if $\hat{u} = 1/2$ and $\hat{D} > 0$, which would correspond to information explicitly provided in transparent concordant treatments, the social learner maximizes the expected payoff by perfectly following the majority.

If $\hat{u} = 1/2$ and $\hat{D} = 0$, which would correspond to information explicitly provided in opaque-prior treatments,

$$
E[\Pi^k] = \pi^k \left[\frac{\psi_L + \psi_H}{2} \right]. \tag{17}
$$

The k-th central moment is thus,

$$
E[(\Pi - E[\Pi])^k] = \sum_{j=0}^{k} \binom{k}{j} (-1)^{k-j} E[\Pi^k] (E[\Pi])^{k-j}. \tag{18}
$$

Equations (17) and (18) are independent of f.

In an open-ended post-experiment questionnaire, we asked social learners if the social information was useful. Tables B and C show a qualitative summary of these responses from the opaque sessions. As the tables show, the majority of subjects explicitly volunteered statements indicating that the information was not useful or helpful. Only a handful explicitly stated that the information was helpful for identifying the optimal urn.

Table B. Qualitative summary of responses to an open-ended question after opaque sessions with priors. The question invited social learners to explain if and perhaps why the information they had in the experiment was useful.

Qualitative answer	Frequency
Does not reveal anything	15
Not helpful	14
Only information available (w/o saying information helpful)	1
Useful in terms of implementing subject's strategy (w/o saying strategy itself was useful)	6
Helpful	1
Helped identify optimal urn	6
Total	43

Table C. Qualitative summary of responses to an open-ended question after opaque sessions without priors. The question invited social learners to explain if and perhaps why the information they had in the experiment was useful.

Qualitative answer	Frequency
Does not reveal anything about winning color	11
Not useful because everything random	6
Not at all useful	9
No answer	1
Useful in terms of implementing subject's strategy (w/o saying strategy itself was useful)	13
Useful	5
Total	45
