# Supplementary material for: The Evolution of Facultative Conformity Based on Similarity
Source: PLoS One. 2016 Dec 21;11(12):e0168551. doi: 10.1371/journal.pone.0168551 (PMC5176289; doi:10.1371/journal.pone.0168551)
Supplement: S1 Instructions — (PDF) [file pone.0168551.s005.pdf]

GENERAL INSTRUCTIONS

[Note to *PLoS ONE* reader: In this example, the winning color for demonstrators was blue. This was not true in all sessions as we counterbalanced this value across sessions.]

To facilitate our explanation, we'll only use the masculine form in these instructions and in the experiment. We certainly speak also to our female participants.

You are about to participate in an experiment at the University of Lausanne. During this experiment, you'll have the possibility of earning money that will be directly transferred to your bank account. The amount of money that you will be able to make could be bigger if:

- You carefully read the instructions
- You follow these instructions to the letter
- You think hard about the decisions you will make

If you have questions while reading the instructions, or during the experiment, do not hesitate to raise your hand to call us. However, **it is strictly forbidden to communicate between participants**. If you do not respect this rule, we will have to exclude you from the experiment without any payment.

Your earnings are calculated in points. At the end of the experiment, the points earned will be converted into Swiss Francs at the following exchange rate:

200 points = 1 CHF

You will also receive a fixed amount of 10 CHF for your participation, independent of your decisions.

The experiment consists of several identical periods, which will be repeated always in the same way. At the end of the session, your payment will be computed as the sum of your earnings in each of these periods.

DETAILED INSTRUCTIONS

In this experiment, participants are divided equally into two groups of about 11 participants each. There will not be any interaction between these two groups.

In each of these groups, there are two types of participants: participants of type A and participants of type B. In particular, each group is composed of 5 type-A participants, and the rest are type-B participants. Each type of participant has a different role, and this role remains the same throughout the experiment. In other words, your type is always the same during the experiment.

***Remark**: Each participant's type, as well as the groups, are randomly determined by the computer at the beginning of the experiment. You will not know your type until the experiment begins. Therefore, please carefully read the explanation about the decisions to be made by both types of participants.*

Each member of your group will face two urns: the LEFT urn and the RIGHT urn. Each urn contains fours balls. One of the two urns contains 3 RED balls and 1 BLUE ball, whereas the other urn contains 1 RED ball and 3 BLUE balls. You will not know which of the two urns contains more red balls than blue balls.

The main decision is to choose one of the two urns. One ball will be randomly drawn from the chosen urn by the computer. Your earnings will be determined by the color of the ball drawn. The color that gives you most points (the winning color) depends on your type.

We will now explain more precisely what decisions each type of participant has to make.

Type- A participants

Before making decisions that will count for their earnings, type-A participants are informed of their type and their winning color. The winning color of type-A participants is always BLUE. This means that, if the ball drawn from the chosen urn is blue, they earn 100 points, whereas if the ball is red, they earn 0 points.

There will be 5 periods. In each period, a type-A participant has to choose one of the two urns: either the RIGHT urn or the LEFT urn. Then, a ball will be drawn from the chosen urn at random by the computer and the participant will be informed of the outcome, as well as the number of points earned.

Given that a blue ball is worth more points to type-A participants than a red ball, the urn with 3 blue balls is preferable to the urn that only contains 1 blue ball. Hence, the urn with 3 blue balls is the “optimal” urn. The participant who chooses the “optimal” urn has 3 out of 4 chances of winning. The participant who chooses the sub-optimal urn has only 1 out of 4 chances of winning.

Type-A participants choose between the two urns 5 consecutive times. They then wait for the decisions of type-B participants.

<u>Type- B participants</u>

(TRANSPARENT, BETWEEN) Before making decisions that will count for their earnings, type-B participants are informed of their type and their winning color. The winning color of type-B participants is always RED (BLUE). This means that, if the ball drawn from the chosen urn is red (blue), they earn 100 points, whereas if the ball is blue (red), they earn 0 points.

(TRANSPARENT, WITHIN) Before making decisions that will count for their earnings, type-B participants are informed of their type and their winning color. The winning color of type-B participants can either be RED or BLUE. If the winning color is RED, they earn 100 points if the ball drawn is red and 0 points if the ball drawn is blue. In contrast, if the winning color is BLUE, they earn 100 points if the ball drawn is blue and 0 points if the ball drawn is red.

Remark: The winning color of type-B participants may or may not be the same as the winning color of type-B participants. Therefore, the optimal urn of type-A participants could be the optimal urn of type-B participants, or it could be the sub-optimal urn of type-B participants.

(OPAQUE, PRIOR) Before making decisions that will count for their earnings, type-B participants are informed of their type, but they are not informed of their winning color. The winning color of type-B participants can either be RED or BLUE, with equal probability. If the winning color is RED, they earn 100 points if the ball drawn is red and 0 points if the ball drawn is blue. In contrast, if the winning color is BLUE, they earn 100 points if the ball drawn is blue and 0 points if the ball drawn is red.

Remark: The winning color of type-B participants may or may not be the same as the winning color of type-B participants. Therefore, the optimal urn of type-A participants could

be the optimal urn of type-B participants, or it could be the sub-optimal urn of type-B participants.

(OPAQUE, NO PRIOR) Before making decisions that will count for their earnings, type-B participants are informed of their type, but they are not informed of their winning color. They earn 100 points if the ball drawn is of the winning color and 0 points if the ball drawn is not of the winning color.

Once the 5 type-A participants have finished, type-B participants are informed of the distribution of decisions in the last (fifth) period. In other words, they are informed of the number of type-A participants that chose the LEFT urn and the number of type-A participants that chose the RIGHT urn in the fifth period.

After seeing this information, a type-B participant has to choose between the same two urns as type-A participants: either the RIGHT urn, or the LEFT urn. Then, the computer draws a ball from the chosen urn at random, adds the number of points earned to the total earnings, puts the ball back in the urn, and draws a ball again. The computer repeats this procedure (draw a ball, add the points to total earnings, put the ball back in the urn) 5 times.

Type-B participants do not see the color of the balls drawn from the chosen urn. Hence, they do not see the number of points earned until the end of the experiment.

Remark*: the number of points earned by each participant depends only on his own decisions. The different types of participants receive different information. A type-A participant never sees what other participants do, but only his earnings after each decision made. A type-B participant sees the final distribution of decisions of type-A participants after 5 periods, but he does not see his earnings after his own decisions. Type-B participants only see the amount earned at the end of the experiment.*

To sum up, this is the sequence of decisions to be made:

1. (TRANSPARENT, BETWEEN & TRANSPARENT, WITHIN) All participants are informed of their type and their winning color.
   (OPAQUE, PRIOR) All participants are informed of their type. The winning color of type-A participants is RED. The winning color of type-B can either be RED or BLUE with equal probability.
   (OPAQUE, NO PRIOR) All participants are informed of their type. The winning color of type-A is RED. Type-B does not know his winning color.

2. Type-A participants choose 5 times one of the two urns and the computer draws every time a ball from the chosen urn. The participant is informed of the outcome and of the number of points earned. In the meantime, type-B participants wait.

3. Type-B participants are informed of the distribution of decisions of type-A participants in the last period. Then, they choose one of the two urns and the computer draws 5 balls with replacement from the chosen urn.

The sequence of decisions described above will be repeated 20 rounds. This means that type-A participants have to choose 100 times (5 decisions in each round) and that type-B participants have to choose 20 times (1 decision in each round). Remember that your type is the same throughout the experiment.

(TRANSPARENT, BETWEEN) It is very important to notice that the optimal urn of each participant may change between the 20 rounds. For example, if the LEFT urn is the optimal urn in the first round, it may be possible that the RIGHT urn is the optimal urn in the second round. However, the winning color remains the same in all 20 rounds.

(TRANSPARENT, WITHIN) It is very important to notice that the optimal urn of each participant as well as the winning color of type-B participants may change between the 20 rounds. For example, if the LEFT urn is the optimal urn in the first round, it may be possible that the RIGHT urn is the optimal urn in the second round. It is also possible that the winning color of type-B participants is RED in the first round, but BLUE in the second round.

(OPAQUE, PRIOR) It is very important to notice that the optimal urn of each participant as well as the winning color of type-B participants may change between the 20 rounds. For example, if the LEFT urn is the optimal urn in the first round, it may be possible that the

RIGHT urn is the optimal urn in the second round. It is also possible that the winning color of type-B participants is RED in the first round, but BLUE in the second round.

(OPAQUE, NO PRIOR) It is very important to notice that the optimal urn of each participant may change between the 20 rounds. For example, if the LEFT urn is the optimal urn in the first round, it may be possible that the RIGHT urn is the optimal urn in the second round.

WHAT WILL YOU DO?

(TRANSPARENT, BETWEEN & TRANSPARENT, WITHIN) At the beginning of each round, you will see on your screen your type and winning color for each type of participant.
(OPAQUE, PRIORS & OPAQUE, NO PRIOR) At the beginning of each round, you will see on your screen your type and the winning color of type-A participants.

This information will be presented on the screen as in Figure 1. Once you have read the information, you will click on the "OK" button at the bottom of the screen.

Fig. 1 : Information concerning type and winning color (of type-A)

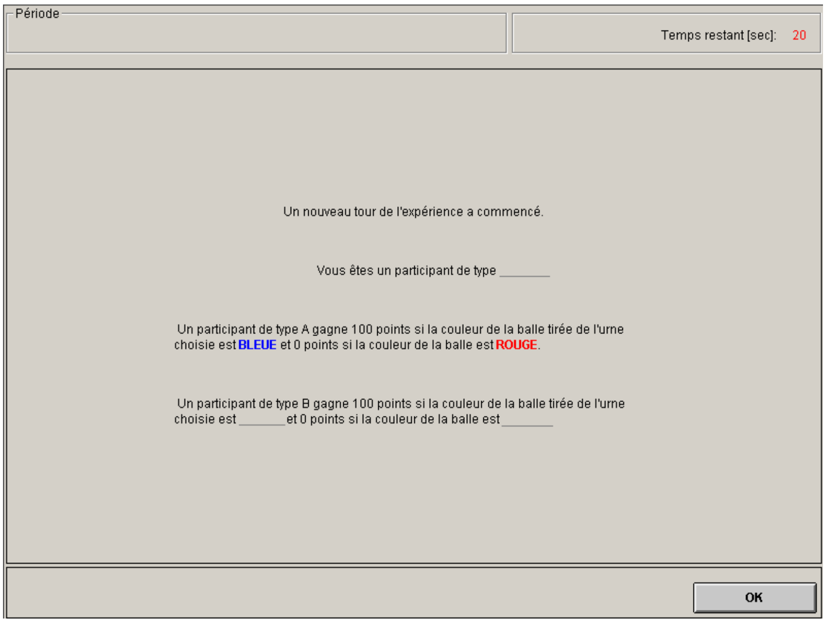

If you are a type-A participant, you will then choose one of the two urns. You will do that with the screen of Figure 2. Once you have made your choice, you will click on the “OK” button at the bottom of the screen.

Fig. 2 : Screen to enter decision (type-A participants)

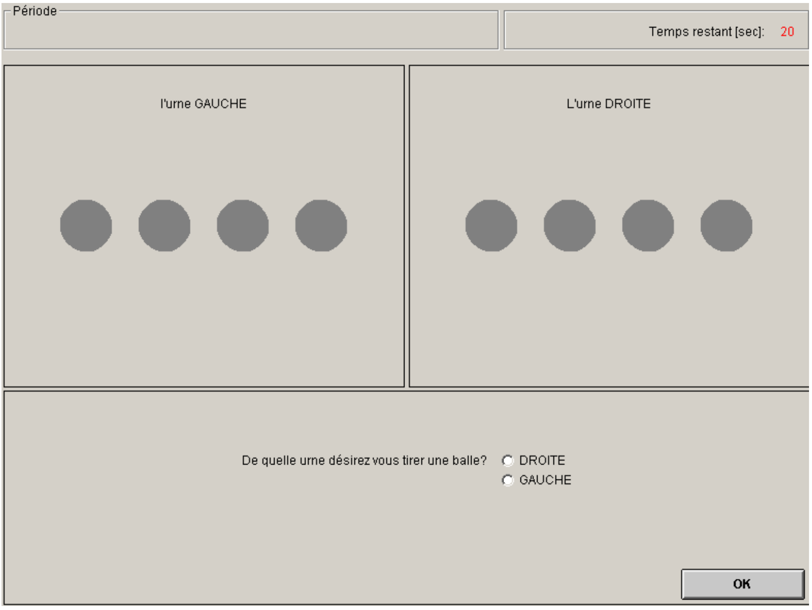

***Remark**: Be careful with the labels of the choices before clicking. The option to select the LEFT urn may appear either <u>above</u> the option to select the RIGHT urn (as in the example of Fig. 2), or <u>below</u> it!*

Next, you will see on the screen your choice, as well as the ball drawn by the computer and the number of points earned. An example of such a screen is presented in Figure 3. Once you have read the information, click on the “OK” button at the bottom of the screen.

Fig.3 : The outcome of my decision (type- A participants)

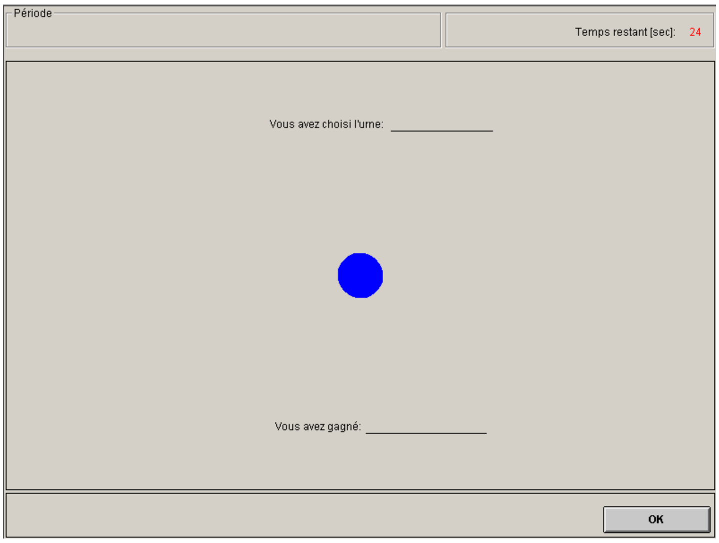

If you are a type-B participant, you wait while the type-A participants are choosing. Afterwards, you'll see the distribution of their decisions in the fifth period, and you will have to choose one of the two urns. You will do that using the screen of Figure 4. Once you have made your choice, you will click on the "OK" button at the bottom of the screen.

Fig.4 : Screen to enter my decision (type-B participants)

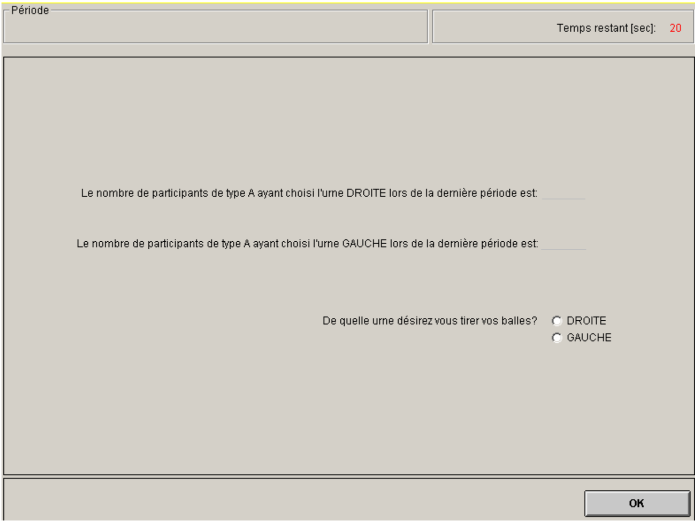

***Remark**: Be careful with the labels of the choices before clicking. The option to select the LEFT urn may appear either below the option to select the RIGHT urn (as in the example of Fig. 4), or above it!*

Finally, the screen of Figure 5 will show you your choice. Once you have read the information, you will click on the "OK" button at the bottom of the screen.

Fig.5 : The outcome of my decision (type-B participants)

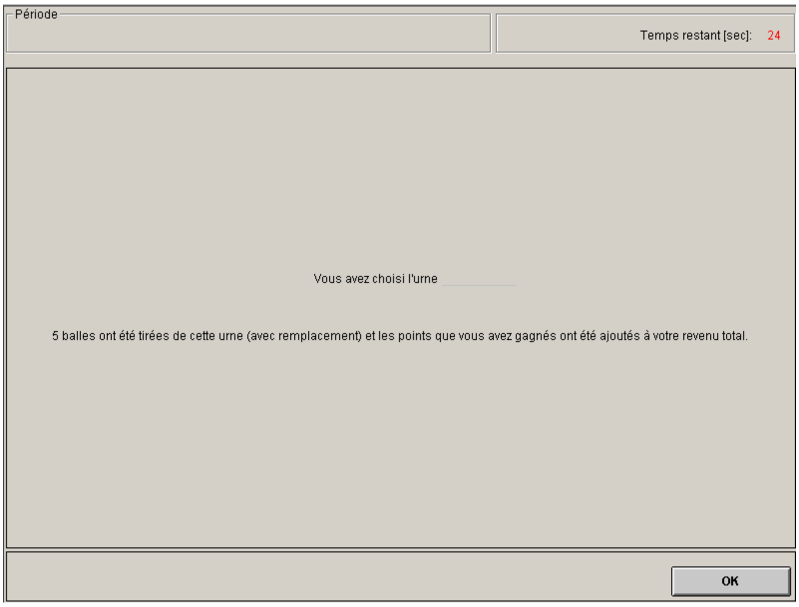

CONTROL QUESTIONS

Before starting with the rounds that will count for your earnings in this experiment, we want to make sure that you and all other participants have correctly understood the decisions to be made. For this, please answer the following questions. **When you have finished, raise your hand so that the assistants come check your answers.**

1) The LEFT urn has the same number of red balls as the RIGHT urn.

a) True b) False

2) The LEFT urn of type-A participants is the same as the LEFT urn of type-B participants.

a) True b) False

3) The winning color is the same for all participants of the same type.

a) True b) False

4) The winning color of type-A participants is always the same as the winning color of type-B participants.

a) True b) False

5) Your type as a participant may change between the rounds.

a) True b) False

6) In the optimal urn, which is the probability of drawing a ball of the winning color?

a) 1/4 b) 1/2 c) 3/4 d) 0.99

7) When a type-A participant makes his decision, does he immediately see the number of points earned?

a) Yes b) No c) Sometimes, but not always

8) When a type-A participant makes his decision, how much does he earn?

(TRANSPARENT, BETWEEN & TRANSPARENT, WITHIN & OPAQUE, PRIOR)

a) He earns 100 points if the ball drawn is RED and 0 point if it is BLUE
b) He earns 100 points if the ball drawn is BLUE and 0 point if it is RED
c) He earns 100 points independent of his choice

(OPAQUE, NO PRIOR)

a) He earns 100 points if the ball drawn is of the winning color and 0 points otherwise.
b) He earns 100 points independent of his choice

9) When a type-B participant makes his decision, does he immediately see the number of points earned?

a) Yes b) No c) Sometimes, but not always

10) When a type-B participant makes his decision, how much does he earn?

a) He earns 100 points if the ball drawn is RED and 0 points if it is BLUE
b) He earns 100 points if the ball drawn is BLUE and 0 points if it is RED
c) He earns 100 points if the ball drawn is of the winning color and 0 points otherwise
d) He earns 100 points independent of his choice

11) Which information is shown to type-B participants?

a) The number of type-A participants who have chosen the LEFT urn and the number of type-A participants who have chosen the RIGHT urn in the fifth period.
b) The number of type-A participants who have chosen the LEFT urn and the number of type-A participants who have chosen the RIGHT urn in the fifth period.
c) The sum of points earned by each type-A participant in the fifth period.
d) The sum of points earned by each type-A participant in the fifth period.
e) No information.
